# Effect of 13 traditional Chinese medicine drug preparations against *Neisseria gonorrhoeae*

**DOI:** 10.3389/fphar.2025.1694041

**Published:** 2025-11-12

**Authors:** Wenfeng Liao, Ke Zhou, Yan Zhang, Yuanqin Huang, Xia Zhang, Yueping Yin, Qian Zhou, Shaochun Chen, Weiyun Li, Wenqi Xu

**Affiliations:** 1 Key Laboratory of Cancer Prevention and Therapy, Department of Urologic Oncology, National Clinical Research Center for Cancer, Tianjin’s Clinical Research Center for Cancer, Tianjin Medical University Cancer Institute and Hospital, Tianjin, China; 2 Tianjin Academy of Traditional Chinese Medicine Affiliated Hospital, Tianjin, China; 3 Taizhou Center for Disease Control and Prevention, Taizhou, China; 4 State Key Laboratory of Green Pesticide, Key Laboratory of Green Pesticide and Agricultural Bioengineering, Ministry of Education, Center for Research and Development of Fine Chemicals, Guizhou University, Guiyang, China; 5 Department of Biology, Hong Kong Baptist University, Hong Kong SAR, China; 6 Hospital for Skin Diseases, Institute of Dermatology, Chinese Academy of Medical Sciences and Peking Union Medical College, Nanjing, China; 7 STD Reference Laboratory, National Center for Sexually Transmitted Diseases Control, Chinese Center for Disease Control and Prevention, Nanjing, China

**Keywords:** antibacterial effect, traditional Chinese medicine (TCM), *Neisseria gonorrhoeae*, *in vitro*, gonorrhea, antimicrobial susceptibility testing (AST)

## Abstract

**Introduction:**

Gonorrhea poses severe health complications, with an estimated 82.4 million new adult infections reported globally in 2020. Currently, ceftriaxone monotherapy remains the first-line treatment in China. However, rising antimicrobial drug resistance necessitates has spurred the urgent need to explore novel therapeutic strategies. This study assessed the antibacterial activity of 13 traditional Chinese medicines (TCMs) against *Neisseria gonorrhoeae* (*N*. *gonorrhoeae*), aiming to identify alternative agents to combat antimicrobial drug resistance.

**Methods:**

The minimum inhibitory concentrations (MICs) of 13 TCMs on 280 *N. gonorrhoeae* isolates were determined using the agar dilution method. The correlation between the MICs of TCMs and those of antibiotics was analyzed using the correlation coefficient (R value). The chemical profiles of TCMs were identified using Gas Chromatography-Mass Spectrometry (GC-MS) and Liquid Chromatography-Tandem Mass Spectrometry (LC-MS/MS). The MICs of representative chemical metabolites on 53 *N. gonorrhoeae* isolates were determined using the agar dilution method.

**Results:**

Coptidis Rhizoma (CR) exhibited the lowest MIC_10_ of ≤0.06 mg/mL and lowest MIC_90_ of ≤0.5 mg/mL. Six TCMs showed no correlation with the MIC values of ceftriaxone, spectinomycin or azithromycin.

**Discussion:**

This study represents the first report about the antibacterial activity of these 13 TCMs against *N. gonorrhoeae*. The results indicate that CR, Phellodendri Chinensis Cortex (PCC), Forsythiae Fructus (FF), Taraxaci Herba (TH), and Scutellariae Radix (SR) exhibited good antibacterial activity against *N. gonorrhoeae*, highlighting their potential as promising therapeutic options for gonococcal infections. In contrast, the MICs of Bupleuri Radix (BR), Cimicifugae Rhizoma (CFR) showed correlations with those of ceftriaxone, azithromycin, and spectinomycin, suggesting that they may be unsuitable for monotherapy of gonococcal infections, given the potential risk of cross-resistance. Nevertheless, further *in vitro* experiments and clinical studies are required to validate these observations.

## 1 Introduction

Gonorrhea, caused by *Neisseria gonorrhoeae* (*N. gonorrhoeae*), is one of the most commonly reported sexually transmitted infections. Left untreated, it can lead to severe reproductive, maternal, and neonatal complications, including infertility, ectopic pregnancy, maternal mortality, and serious ocular infections (even blindness) in newborns, while also increasing the risk of HIV transmission (Hu H. et al., 2022; Liu J. et al., 2022; Su et al., 2022; Suyama and Akiyama, 2022). These complications, combined with shrinking treatment options and prolonged therapeutic courses, have escalated the medical burden on individuals and healthcare systems globally. According to the World Health Organization (WHO), there were 87 million new gonorrhea cases worldwide in 2016 (Rowley et al., 2019), up from 78 million in 2012 (Newman et al., 2015). In China, the incidence rate rose from 7.36 per 100,000 in 2015 to 10.06 per 100,000 in 2017, before declining to 8.45 per 100,000 in 2019, resulting in an overall average annual increase of 3.51% (Zheng et al., 2020). Therefore, this trend underscores the urgent need for enhanced efforts to combat gonorrhea.

Antibiotics remain the cornerstone of gonorrhea treatment. However, due to inappropriate use and inadequate monitoring, *N. gonorrhoeae*’s susceptibility to both previously and currently recommended antibiotics has declined. The persistent emergence and global spread of drug-resistant strains have become critical public health challenges (Unemo and Shafer, 2011; Unemo, 2015; Huang et al., 2024; Khatun et al., 2024; Liu et al., 2024; Dai et al., 2025; Tama et al., 2025). Ceftriaxone and azithromycin are recommended as first-line therapies from the United States (Abrams and Trees, 2017), Europe (Merrick et al., 2022), Australia (Bavinton et al., 2022) and WHO. In China, ceftriaxone monotherapy is currently recommended for uncomplicated gonococcal infections per existing guidelines. However, *N. gonorrhoeae* has developed drug resistance to ceftriaxone and all previously used antimicrobial therapies, including azithromycin (Dos Santos et al., 2022; Goytia and Wadsworth, 2022; Visser et al., 2022), drastically narrowing available treatment options. Therefore, there is an urgent need to identify alternative therapeutic agents and develop new drugs.

Amid rising gonococcal resistance, efforts to develop new antimicrobial drugs (e.g., tigecycline and meropenem) have been pursued, but such development and deployment require substantial financial investment and time. Exploring alternative medicines thus offer a more feasible and cost-effective approach. TCM-based botanical drug therapies are gaining global recognition (Ji et al., 2024; Tama et al., 2024; Wang et al., 2025; Xu et al., 2025), with some TCMs demonstrating promising efficacy against various pathogens, including potential activity against *N. gonorrhoeae* (Wong et al., 2010; Guo et al., 2016; Ming et al., 2022; Yagüe et al., 2022; Hussein et al., 2025; Tahir et al., 2025). However, their specific antibacterial activity against *N. gonorrhoeae* remains poorly characterized.

In this work, we for the first time evaluated the *in vitro* antibacterial activity of TCMs against *N. gonorrhoeae*, yielding foundational data to support their potential as alternative antimicrobials for clinical gonorrhea treatment. Additionally, we performed qualitative analysis of chemical metabolites in TCM capsules using GC-MS and LC-MS/MS. Subsequently, we evaluated the *in vitro* antibacterial activity of representative metabolites from these TCMs against *N. gonorrhoeae*. This work not only seek to identify novel strategies for combating gonorrhea but also provides methodological references for related research.

## 2 Materials and methods

### 2.1 Traditional Chinese medicines, clinical antibiotics and selected representative metabolites

The species name, family, and Latin pharmacological name of 13 TCMs studied are listed below.

1. *Portulaca oleracea* L. [Portulacaceae; Portulacae Herba (PH)]
2. *Patrinia scabiosifolia* Fisch. ex Trevir. [Caprifoliaceae; Patrinia Scabioseafolia (PS)]
3. *Dioscorea septemloba* Thunb. [Dioscoreaceae; Dioscoreae Spongiosae Rhizoma (DSR)]
4. *Coptis chinensis* Franch. [Ranunculaceae; Coptidis Rhizoma (CR)]
5. *Bupleurum chinense* DC. [Apiaceae; Bupleuri Radix (BR)]
6. *Phellodendron chinense* Schneid. [Rutaceae; Phellodendri Chinensis Cortex (PCC)]
7. *Forsythia suspensa* (Thunb.) Vahl. [Oleaceae; Forsythiae Fructus (FF)]
8. *Taraxacum mongolicum* Hand. - Mazz. [Asteraceae; Taraxaci Herba (TH)]
9. *Scutellaria baicalensis* Georgi [Lamiaceae; Scutellariae Radix (SR)]
10. *Cinnamomum cassia* Presl. [Lauraceae; Cinnamomi Cortex (CC)]
11. *Cimicifuga foetida* L [Ranunculaceae; Cimicifugae Rhizoma (CFR)]
12. *Atractylodes Lancea* (Thunb.) DC. [Asteraceae; Atractylodis Rhizoma (AR)]
13. *Sophora flavescens* Ait. [Leguminosae; Sophorae Flavescentis Radix (SFR)]

13 TCMs were sourced from Tianjin Academy of Traditional Chinese Medicine Affiliated Hospital. These were single-flavor TCM granule decoction pieces manufactured by Sichuan Neo-Green Pharmaceutical Technology Development Co., Ltd. The TCM granules were prepared following standard decoction protocols, with various modern analytical techniques employed as quality indicators. The production process involved parameter-optimized extraction, incorporating β-cyclodextrin (β-CD)—a cyclic oligosaccharide—wherein drug molecules are fully or partially encapsulated to form non-covalently bound complexes. Subsequent steps included solid-liquid separation, vacuum low-temperature concentration, spray-drying, and dry granulation (granules formed via physical extrusion through a flat pressing cylinder). Active ingredients were formulated into granules to facilitate quantitative application, and this preparation method has been granted a national patent. The relevant parameters for the preparation and identification of 13 TCM granules under the framework of national standard are briefly summarized and presented in Supplementary Table S1. Clinical antibiotics (spectinomycin, ceftriaxone, cefixime, and azithromycin) and 18 selected representative metabolites from these TCMs were purchased from Sigma Aldrich and accordance with United States Pharmacopeia (USP) grade standards.

### 2.2 Gonococcal isolates

A total of 280 clinical gonococcal isolates were collected from four Chinese provinces: Guangdong (n = 71), Liaoning (n = 73), Hunan (n = 83), and Hainan (n = 53). These isolates were acquired via the China Gonococcal Resistance Surveillance Program (China-GRSP) during 2020 and 2021. This study was approved by the Medical Ethics Committee at the Institute of Dermatology, Chinese Academy of Medical Sciences and Peking Union Medical College, as well as the National Center for Sexually Transmitted Disease Control (2014-LS-026).

All isolates were obtained from urogenital secretions of confirmed gonorrhea patients. They were inoculated, identified, preserved, and transferred according to previously described methods (Yin et al., 2018). Prior to antimicrobial susceptibility testing, all strains were stored in skim milk in a deep freezer at −80 °C. Quality control was ensured using ten WHO *N. gonorrhoeae* reference isolates (G, J, K, L, O, P, X, Y, Z and V) and ATCC 49226.

This study complied with the Declaration of Helsinki guidelines. All participants provided informed consent prior to enrollment. Eligible participants were aged ≥18 years and has signed informed consent to provide urine, vaginal and rectal swabs.

### 2.3 Antimicrobial susceptibility testing of TCMs and selected representative metabolites

The antimicrobial susceptibility of isolates to the 13 aforementioned TCMs and selected representative metabolites was assessed via the WHO standard agar dilution method (Wiegand et al., 2008; Balouiri et al., 2016; Riedel et al., 2019). Concurrently, the MICs of ceftriaxone, cefixime, spectinomycin, and azithromycin were determined. Specifically, isolates were cultured from frozen stocks onto selective Thayer-Martin (TM) medium and subsequently sub-cultured on Gonococcal (GC) medium agar supplemented with hemoglobin and IsoVitaleX Enrichment (BD Diagnostics, Oxford, England) at 36 °C with 5%–10% CO_2_ for 18–20 h in the Sexually Transmitted Disease (STD) laboratories. Colonies were scraped to prepare suspensions of 10^7^ colony-forming units (CFU)/mL. Using a multipoint inoculator (10^4^ CFU per point), suspensions was inoculated onto antibiotic-containing medium (GC agar base supplemented with 10% defibrinated sheep blood; Nanjing Bianzhen Biotechnology, Nanjing, China) on a 9 cm diameter plate. The plate was then cultured at 36 °C with 5%–10% CO_2_ for 18–24 h to assess *N. gonorrhoeae* growth at varying concentrations of the antibiotics, 13 TCMs or selected representative metabolites.

The concentrations for the TCMs were as follows: for POL, DSR, BCDC, FS, TMHM, CC, CFL, SFR, the concentrations were 4, 8, 16, 32, and 64 mg/mL; for PS, they were 4, 8, 16, and 32 mg/mL; for CR and PCS, the concentrations were 0.06, 0.125, 0.25, 0.5, 1, 2 mg/mL, and for AL and SB, they were 8, 16, 32 and 64 mg/mL. For the three antibiotics, the concentrations were 2, 4, 8, 16 and 32 mg/L for spectinomycin; 0.008, 0.015, 0.03, 0.06, 0.125, 0.25, 0.5, and 1 mg/L for ceftriaxone; and 0.06, 0.125, 0.25, 0.5, 1, 2, and 4 mg/L for azithromycin. The lowest concentration of the antibiotic that inhibited isolate growth was defined as MIC.

Currently, there are no standardized criteria for TCMs regarding *N. gonorrhoeae* treatment as established by the European Committee on Antimicrobial Susceptibility Testing (EUCAST) and the Clinical and Laboratory Standards Institute (CLSI). Quality assurance was maintained using ten WHO *N. gonorrhoeae* reference isolates (G, J, K, L, O, P, X, Y, Z and V) and ATCC 49226.

### 2.4 Data analysis

The MIC of the reference isolate was considered acceptable if it matched the standard or varied within one MIC dilution step; thus, batch data meeting this criterion were included in the analysis. The distribution of MICs for the 13 TCMs against the collected *N. gonorrhoeae* isolates was characterized. To date, no standardized criteria for TCMs in *N. gonorrhoeae* testing have been established by EUCAST or CLSI. The correlation between the TCM MICs and those of ceftriaxone, spectinomycin or azithromycin was analyzed via linear regression of log_2_-transformed MIC data from the 280 isolates. Cross-resistance was defined as an absolute correlation coefficient (R) > 0.3, with statistical significance set at p < 0.05. Specifically, weak, moderate, and strong correlations were categorized as R = 0.3–0.5, 0.5–0.8, and 0.8–1.0, respectively. Statistical analysis was performed using SPSS software (IBM, New York, USA) and Excel (Microsoft, Washington, USA). Figures were generated using R (GNU system).

### 2.5 Gas chromatography-mass spectrometry (GC-MS) analysis

GC-MS analysis was conducted at Hong Kong Baptist University. Samples were ground to a fine powder using a mortar and pestle. Exactly 80 mg of powder was transferred to a glass tube and extracted with 1 mL hexane (containing 6.6 ng of the internal standard naphthalene), followed by vertexing and sonication at room temperature for 30 min. The supernatant, containing extracted metabolites, was transferred to a fresh glass tube and concentrated to ≈200 *µ*L under nitrogen prior to injection into gas chromatography-mass spectrometry (GC-MS). For metabolite profiling, three biological replicates were used per TCM (Krause et al., 2021).

### 2.6 Liquid chromatography-tandem mass spectrometry (LC-MS/MS) analysis

LC-MS/MS analysis was performed at Shanghai Applied Protein Technology Co. (Shanghai, China). Samples were ground into powder at room temperature. Exactly 100 mg of powder was weighed and transferred to a 1.5 mL centrifuge tube. Next, 1 mL of 70% methanol aqueous was added. The mixture was thoroughly vortexed for 30 s, then subjected in a water bath (power: 600 W, frequency: 40 kHz) for 30 min. After sonication, the sample was centrifuged at 16,000 × g for 10 min at 4 °C. The supernatant was transferred to a 96-well protein filtration plate and filtered under positive nitrogen pressure. The filtrate was then moved to a 2 mL microcentrifuge tube and vacuum dried. After drying, 400 *μ*L of 40% methanol aqueous was added to reconstitute the residue. The mixture was vortexed for 30 s and recentrifuged at 16,000 × g for 10 min at 4 °C, and the supernatant was collected for further analysis.

Samples were analyzed using a Vanquish UHPLC system (Thermo Fisher Scientific, Bremen, Germany) coupled with an ACQUITY UPLC HSS T3 column (2.1 mm × 100 mm, 1.8 *µ*m) for separation. The column temperature was maintained at 35 °C, with a flow rate of 0.3 mL/min. The mobile phase consisted of solvent A: 0.1% formic acid in water and B: 0.1% formic acid in acetonitrile, with gradient elution applied.

A Q-Exactive HFX mass spectrometer, interfaced with the UHPLC system, was used to acquire both primary (MS1) and secondary (MS2) mass spectra. Electrospray ionization (ESI) was performed in both positive (ESI+) and negative (ESI−) modes. The spray voltage was set to 3800 V (ESI+) and 3500 V (ESI−), with sheath gas and auxiliary gas pressures at 45 arb and 20 arb, respectively. The ion transfer tube temperature was maintained at 320 °C, and the ion source temperature was 350 °C.

Detection was conducted in full scan/data-dependent acquisition (Full-MS/dd-MS^2^) mode, with MS1 and MS2 resolutions set to 60,000 and 15,000, respectively. The top 10 MS1 ions were selected for MS/MS analysis, using normalized stepped collision energies of 20, 40, and 60 eV. The MS1 scanning range (m/z, mass-to-charge ratio) was 90–1,300.

## 3 Results

### 3.1 Antimicrobial susceptibility of 280 *Neisseria gonorrhoeae* isolates

Among the 280 isolates, the number of specific strains with effective MIC values for each TCM is shown in Table 1. All isolates demonstrated effective MICs for ceftriaxone and azithromycin. The range, MIC_10_, and MIC_90_ of the 13 TCMs are also presented in Table 1. CR exhibited the lowest MIC_10_ of ≤0.06 mg/mL, followed by PCC with an MIC_10_ of 0.25 mg/mL. The MIC_10_ of FF was slightly higher at 4 mg/mL. The MIC_10_ values for PH, TH, and CFR were 8 mg/mL, while SR had a comparable MIC_10_ value of ≤8 mg/mL. PS, SFR, and CC showed the same MIC_10_ of 16 mg/mL, and both DSR and AR had an MIC_10_ of 32 mg/mL. Lastly, BR exhibited the highest MIC_10_ of 64 mg/mL.

**TABLE 1:** Antimicrobial susceptibility testing results for clinical *Neisseria gonorrhoeae* isolates.

Traditional Chinese medicine	Isolates	MIC (mg/mL)		
		Range	MIC_10_	MIC_90_
Portulacae Herba (PH)	138	8–16	8	16
Patrinia Scabiosaefolia (PS)	71	8-≥32	16	16
Dioscoreae Spongiosae Rhizoma (DSR)	142	16->64	32	64
Coptidis Rhizoma (CR)	71	≤0.06–1	≤0.06	0.5
Bupleuri Radix (BR)	70	32->64	64	64
Phellodendri Chinensis Cortex (PCC)	67	0.125–1	0.25	1
Taraxaci Herba (TH)	139	≤4->64	8	16
Forsythiae Fructus (FF)	67	2–16	4	16
Scutellariae Radix (SR)	209	≤8->64	≤8	16
Cinnamommi Cortex (CC)	70	16->64	16	32
Cimicifugae Rhizoma (CFR)	70	8->64	8	16
Atractylodis Rhizoma (AR)	70	32->64	32	64
Sophorae Flavescentis Radix (SFR)	70	8->64	16	32
Ceftriaxone	280	≤0.008–2	0.016	0.06
Azithromycin	280	≤0.03–16	0.125	0.5
Spectinomycin	280	4–16	8	16

The MIC_90_ of the TCMs was generally higher than the MIC_10_, with the exception of PS and BR, for which the two values remained unchanged. The MIC_90_ of CR was still the lowest at ≤ 0.5 mg/mL, followed by PCC with an MIC_90_ of 1 mg/mL. The MIC_90_ values for PH, PS, TH, FF, SR, and CFR were all 16 mg/mL, while SFR and CC shared an MIC_90_ of 32 mg/mL. AR, DSR, and BR exhibited the highest MIC_90_ of 64 mg/mL.

### 3.2 Correlation between MICs of TCMs and different antibiotics

To assess potential cross-resistance of *N. gonorrhoeae* to various drugs, the correlation between 13 TCMs and the antibiotics spectinomycin, ceftriaxone, and azithromycin was analyzed (Table 2), along with their R values and P-values. Notably, correlations between ceftriaxone and TCMs were only observed for BR and CFR, with correlation coefficients of R = 0.42 and 0.32, respectively (P < 0.05).

**TABLE 2:** Correlation between MICs of TCMs and antibiotics.

Traditional Chinese medicine	Antibiotics		
	Ceftriaxone one	Azithromycin	Spectinomycin
Portulacae Herba (PH)	−0.07	0.11	0.03
Patrinia Scabiosaefolia (PS)	−0.07	0.04	0.06
Dioscoreae Spongiosae Rhizoma (DSR)	0.08	0.41*	−0.15
Coptidis Rhizoma (CR)	0.12	0.20	0.12
Bupleuri Radix (BR)	0.42*	0.31^*^	0.33*
Phellodendri Chinensis Cortex (PCC)	0.17	0.21	−0.06
Taraxaci Herba (TH)	0.04	0.24^*^	0.11
Forsythiae Fructus (FF)	0.08	0.03	−0.10
Scutellariae Radix (SR)	0.23*	0.35*	−0.32*
Cinnamommi Cortex (CC)	0.03	0.36*	0.33*
Cimicifugae Rhizoma (CFR)	0.32*	0.34*	0.35*
Atractylodis Rhizoma (AR)	0.08	0.42*	0.36*
Sophorae Flavescentis Radix (SFR)	0.26*	0.45*	0.41*

*: P < 0.05.

**FIGURE 1:**
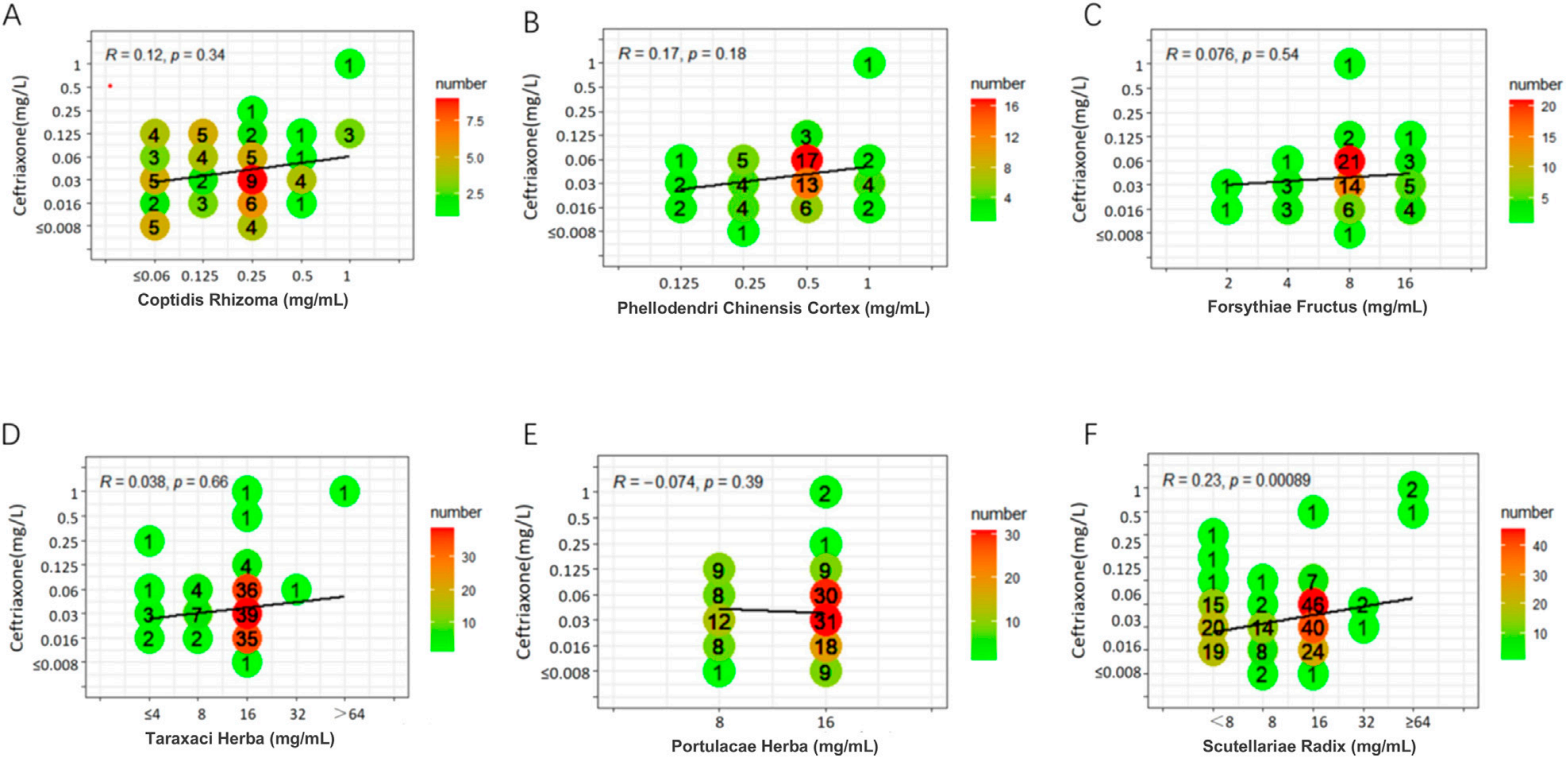
Six scatter plots **(A-F)** analyzing the correlation between various herbal extracts (Coptidis Rhizoma, Phellodendri Chinensis Cortex, Forsythiae Fructus, Taraxaci Herba, Portulacae Herba, Scutellariae Radix) and ceftriaxone concentrations. Each plot shows R and p-values, with color gradients representing numerical frequency. A line of best fit appears in each plot.

**FIGURE 2:**
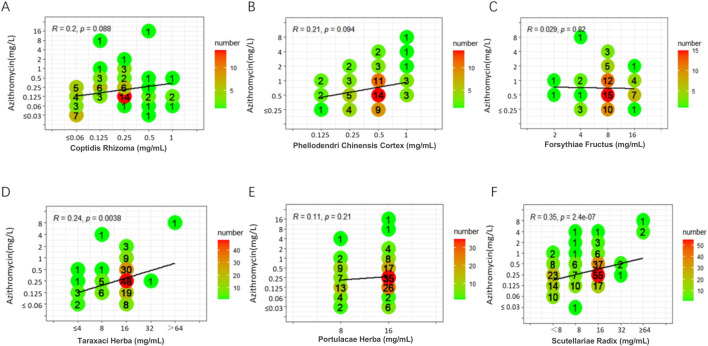
Six scatter plots **(A-F)** show the correlation between azithromycin concentration and six different herbal components, each subplot representing a different herb: Coptidis Rhizoma, Phellodendri Chinensis Cortex, Forsythiae Fructus, Taraxaci Herba, Portulacae Herba, and Scutellariae Radix. The plots display correlation coefficients (R) and p-values. The data points are marked with numbers indicating their frequency, with a color gradient from green to red showing increasing numbers. The trend lines in each plot represent the correlation between the herb concentrations and azithromycin levels.

**FIGURE 3:**
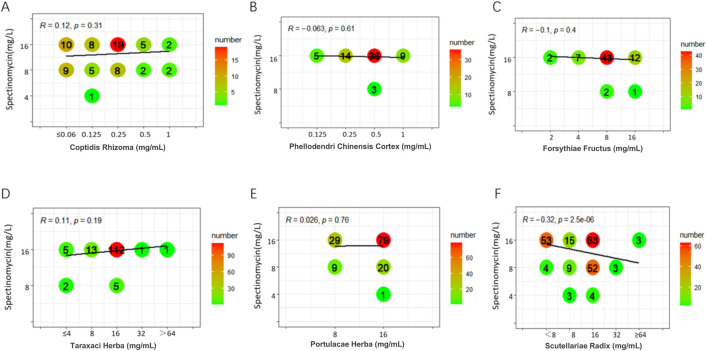
Six graphs display the correlation between Spectinomycin concentration and various herbal extracts: Coptidis Rhizoma **(A)**, Phellodendri Chinensis Cortex **(B)**, Forsythiae Fructus **(C)**, Taraxaci Herba **(D)**, Portulacae Herba **(E)**, and Scutellariae Radix **(F)**. Each graph is labeled with an “R” value and “p” value, indicating the strength and significance of the correlation. The bubble sizes represent the number of samples, with a color gradient from green to red, indicating increasing numbers.

Weak positive correlations were found between azithromycin and seven TCMs (DSR, BR, SR, CC, CFR, AR, and SFR), with R values ranging from 0.31 to 0.45 (P < 0.05). Additionally, a weak positive correlation was observed between spectinomycin and four TCMs (CC, BR, CFR, and SFR), with R values ranging from 0.33 to 0.41 (P < 0.05). In contrast, a weak negative correlation was noted between SR and spectinomycin, which was inconsistent with the above results, yielding R = - 0.32 (P < 0.05). Other drug interaction analyses did not reveal any significant correlations (Figures 1–3; Supplementary Figures S1-S7).

### 3.3 Results of the GC-MS analysis

**FIGURE 4:**
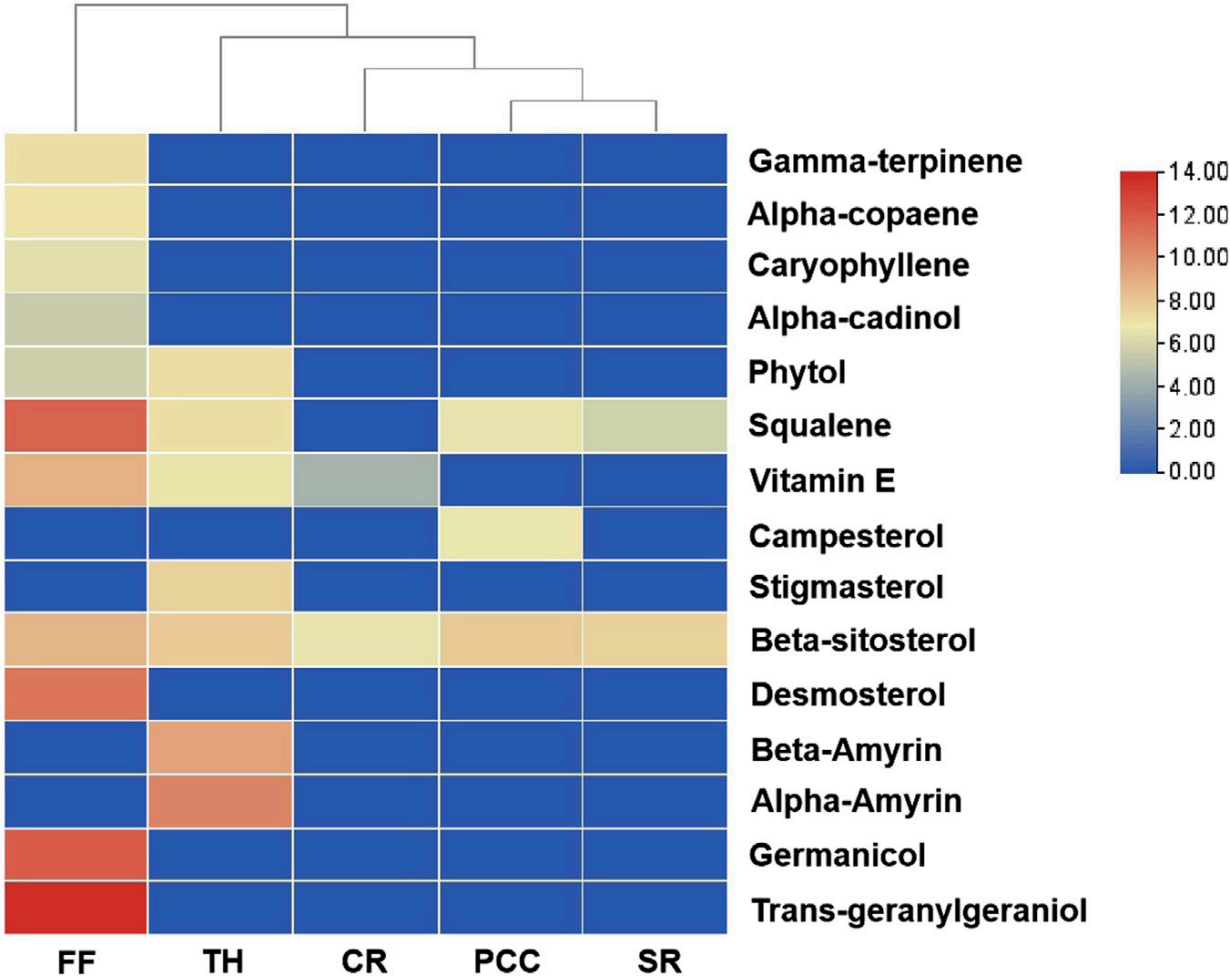
Heatmap of chemical metabolites from five traditional Chinese medicines. FF, Forsythiae Fructus; TH, Taraxaci Herba; CR, Coptidis Rhizoma; PCC, Phellodendri Chinensis Cortex; SR, Scutellariae Radix.

**FIGURE 5:**
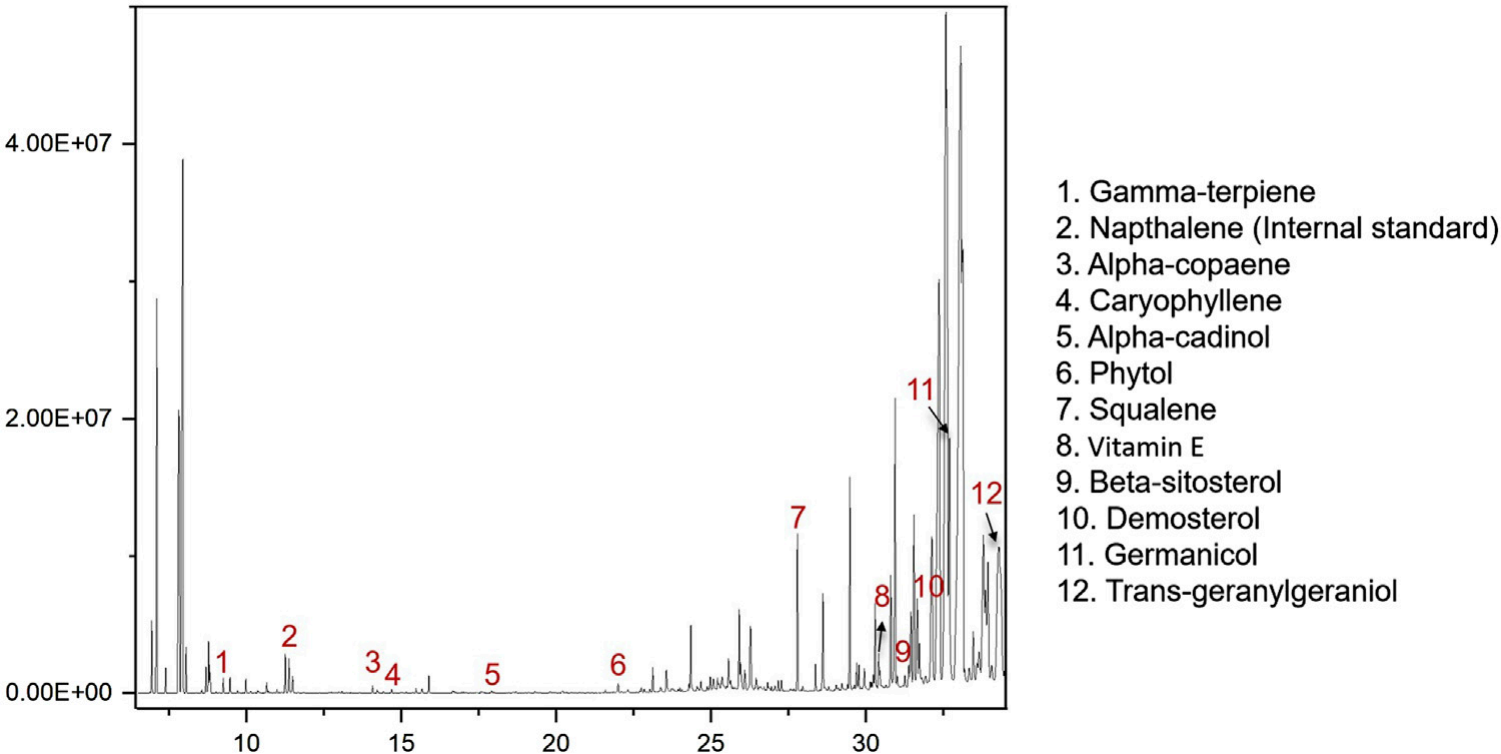
Representative chromatograph of metabolites from Forsythiae Fructus.

**FIGURE 6:**
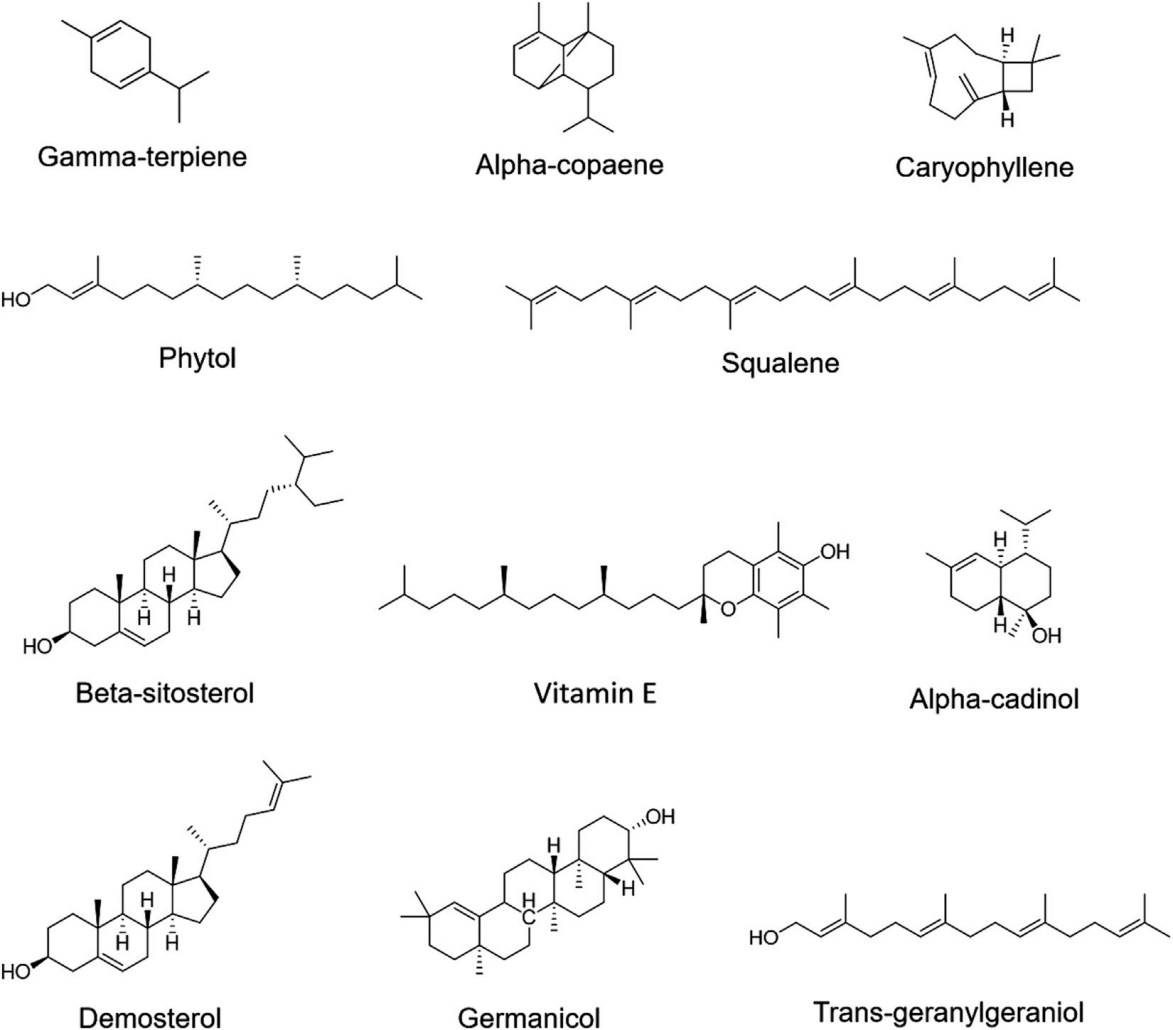
Metabolites and structural formulas of Forsythiae Fructus.

To identify active metabolites with potential antibacterial effects, five TCMs (CR, PCC, TH, FF, and SR) with low MIC values as noted in Section 3.1, were analyzed via GC-MS. The results are presented in Figure 4. A total of 15 metabolites were detected, with all five TCMs containing β-sitosterol. Additionally, squalene was detected in four TCMs (PCC, TH, FF, and SR), vitamin E in three TCMs (CR, TH, and FF), and phytol in two TCMs (FF and TH). Among these, FF contained the largest number of detected metabolites, including gamma-terpiene, alpha-copaene, caryophyllene, alpha-cadinol, phytol, squalene, beta-sitosterol, vitamin E, demosterol, germanicol, and trans-geranylgeraniol. The representative chromatograph for FF is shown in Figure 5, and the chemical structural formulas these metabolites are illustrated in Figure 6.

### 3.4 Results of the LC-MS/MS analysis

**FIGURE 7:**
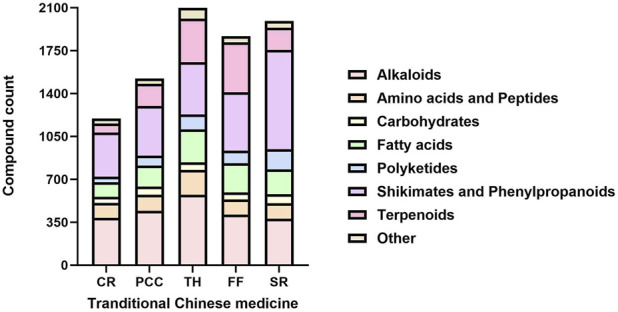
Stacked bar charts depicting the count distribution of metabolites categorized by metabolic pathways across five traditional Chinese medicines (CR, PCC, TH, FF, and SR). CR, Coptidis Rhizoma; PCC, Phellodendri Chinensis Cortex; TH, Taraxaci Herba; FF, Forsythiae Fructus; SR, Scutellariae Radix.

**FIGURE 8:**
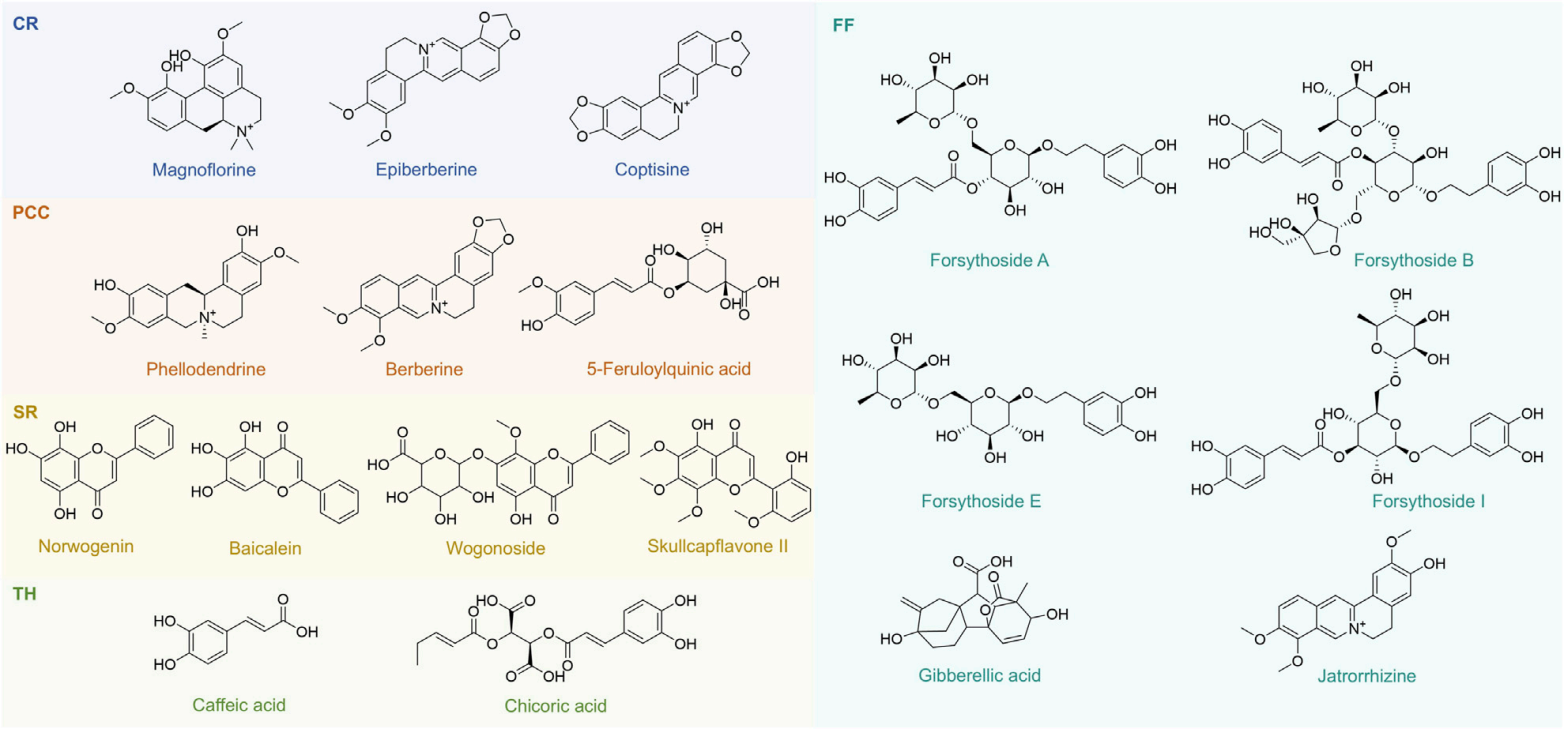
18 Representative metabolites from CR, PCC, SR, TH and FF. CR, Coptidis Rhizoma; PCC, Phellodendri Chinensis Cortex; SR, Scutellariae Radix; TH, Taraxaci Herba; FF, Forsythiae Fructus.

To identify representative metabolites in the tested TCMs, we performed LC-MS/MS analysis on five TCMs (CR, PCC, TH, FF, and SR) with low MIC values mentioned in Section 3.1. The base peak chromatograms (BPCs) of the two most active TCMs in positive ion mode and negative ion mode are shown in Supplementary Figures S8-S9, and the peak-labeled identification results of metabolites in the BPCs are presented in Supplementary Tables S2-S3. Among these TCMs, TH contained the highest number of metabolites (2,098), followed by SR (1,992), FF (1,867), PCC (1,522) and CR (1,197). These metabolites—including alkaloids, carbohydrates, fatty acids, polyketides, terpenoids, phenylpropanoids, and other phytochemicals—were putatively identified via library alignment (Figure 7). Notably, several high-concentration metabolites detected in this analysis have been previously reported to exist in the botanical drug metabolites of TH, SR, FF, PCC, and CR formulations. For example, berberine (a major alkaloid) is known to be abundant in CR, while phellodendrone (a triterpenoid) is a characteristic metabolite of PCC. Several high-concentration metabolites specific to each TCM are presented in Figure 8, and the antibacterial activity of these metabolites will be further verified.

### 3.5 Antimicrobial activity of the selected representation metabolites

**FIGURE 9:**
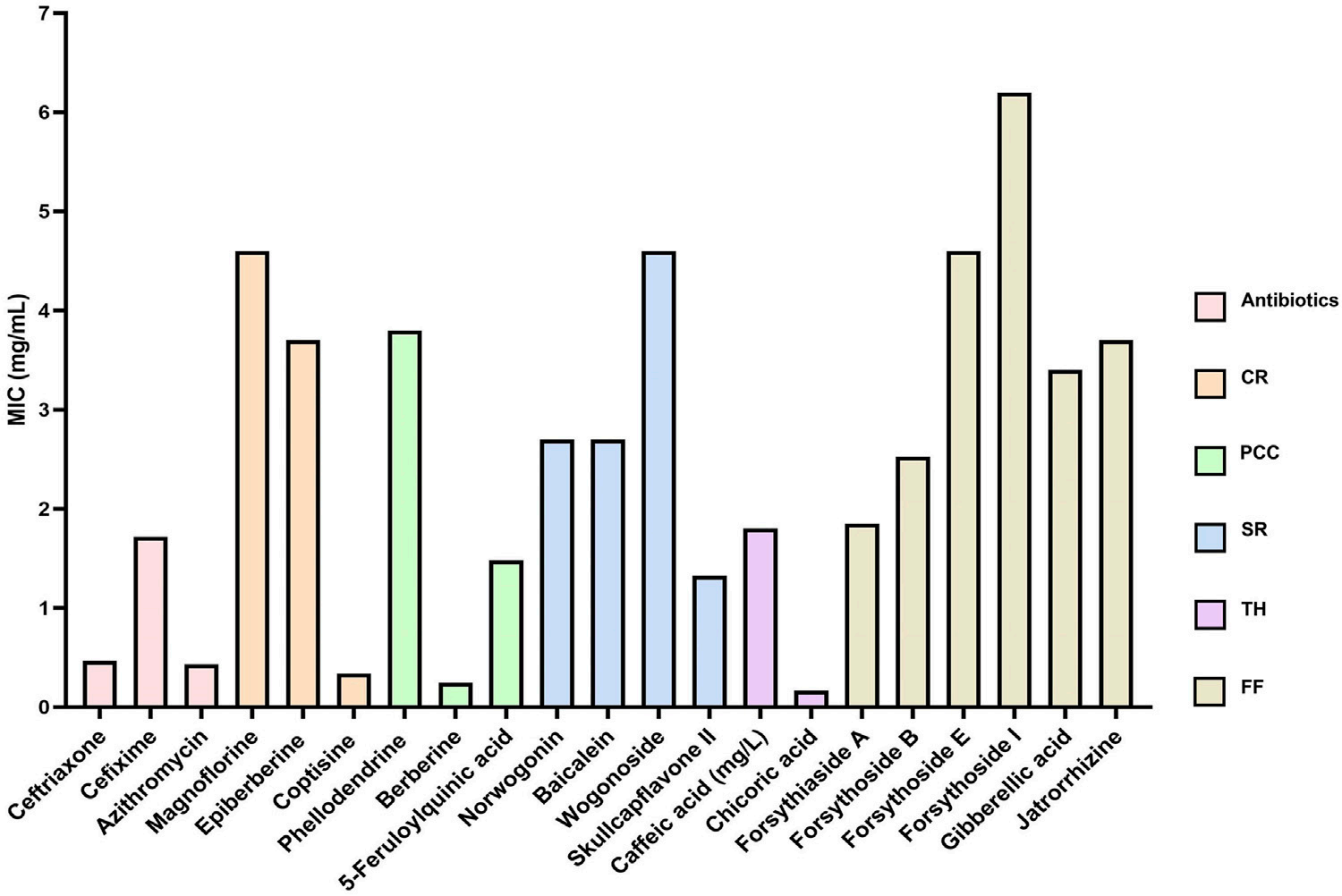
The average MIC value of representative metabolites in antibiotics, CR, PCC, SR, TH and FF. CR, Coptidis Rhizoma; PCC, Phellodendri Chinensis Cortex; SR, Scutellariae Radix; TH, Taraxaci Herba; FF, Forsythiae Fructus.

To identify the antibacterial metabolites in the five aforementioned TCMs with relatively strong antibacterial effects, we selected and purchased 18 representative metabolites (Figure 8) based on the LC-MS/MS results, and assessed their antibacterial activity (Supplementary Table S4). The results are presented in Figure 9, which displays the average MIC value of representative metabolites in antibiotics, CR, PCC, SR, TH, and FF. A lower MIC indicates stronger antibacterial activity against the target strain.

Among the antibiotics, ceftriaxone, cefixime, and azithromycin all exhibited MICs below 2 mg/mL, demonstrating potent antibacterial activity against the target strain. Notably, azithromycin showed the lowest MIC (approximately 0.5 mg/mL), reflecting its relatively superior antibacterial capacity, consistent with its clinical broad - spectrum activity against Gram-positive bacteria and atypical pathogens. For CR, among the alkaloids (magnoflorine, epiberberine, and coptisine), magnoflorine and epiberberine had relatively high MICs (>4 mg/mL) and weak antibacterial activity, whereas coptisine displayed a markedly lower MIC (near 0.5 mg/mL). This discrepancy is closely associated with their molecular structures: the conjugated quaternary ammonium alkaloid structure of coptisine facilitates disruption of the bacterial cell membrane, while the polar groups in magnoflorine may diminish its antibacterial efficacy. In PCC, berberine (the core antibacterial metabolite, as reported in the literature) exhibited a significantly lower MIC (<1 mg/mL) compared to phellodendrine, exerting its effect by inhibiting bacterial DNA gyrase and interfering with metabolic pathways. Among the metabolites of SR (norwogonin, baicalein, wogonoside, and skullcapflavone II), skullcapflavone II had the lowest MIC (∼1.2 mg/mL), implying that this flavonoid may exert strong antibacterial potential by inhibiting bacterial protein synthesis. For TH, chlorogenic acid (with an MIC near 0.5 mg/mL) demonstrated better antibacterial activity than caffeic acid, likely due to the synergistic effects of its antioxidant property and disruption of the bacterial cell wall. In FF, forsythoside E and forsythoside I showed significantly higher MICs (>6 mg/mL and >4 mg/mL, respectively) and weak antibacterial activity, while forsythoside A and forsythoside B had lower MICs (two to three mg/mL). This marked variation in antibacterial activity among metabolites of FF may be attributed to the strain tolerance toward glycoside-based metabolites.

## 4 Discussion

Of the 13 TCMs, six TCMs (CR, PCC, FF, TH, PH and PS) exhibited no correlation with the MIC values of ceftriaxone, spectinomycin, and azithromycin. Among these, CR and PCC showed the most potent activity, with low MIC_90_ values of 0.5 mg/mL and 1 mg/mL, respectively. Their active metabolites are primarily composed of protoberberine alkaloids (e.g., berberine and magnoflorine), a finding consistent with previous studies (Si et al., 2021; Chen et al., 2022). Previous research has demonstrated that these alkaloids possess broad-spectrum antibacterial activity, effective against both Gram-positive and Gram-negative bacteria (Liang J. et al., 2022). Their mechanism of action may involve inhibiting bacterial carbohydrate, nucleic acid, and protein metabolism (Liu et al., 2023). Berberine—a natural isoquinoline alkaloid extracted from *Coptis chinensis* Franch. — has reported antimicrobial activity against fungi, viruses, *chlamydia*, and bacteria, including *N. gonorrhoeae* (Zhang et al., 2021; Noites et al., 2022). Prior studies have shown that berberine can inhibit the growth of penicillinase-producing *N. gonorrhoeae* isolates, regardless of their susceptibility to spectinomycin (Rouquette-Loughlin et al., 2018; Zheng et al., 2020). The presence of these potent alkaloids likely underpins their superior activity observed in this study. FF, TH, PH and PS had the same MIC_90_ value of 16 mg/mL, but different in their MIC_10_ gradients, with values of 4, 8, 8 and 16 mg/mL, respectively. FF has garnered significant attention due to its remarkable pharmacological activity (Jing et al., 2022). Forsythiaside A, the main active metabolite of phenylethanol glycosides we detected in FF, exhibits a strong inhibitory effect against *Staphylococcus aureus* (*S. aureus*) and methicillin-resistant *S. aureus* (Zhang et al., 2018). Moreover, the inhibitory effect of which combined with cefazolin or vancomycin on *S*. *aureus* and methicillin-resistant *S*. *aureus* has been shown to be additive (Gong et al., 2021). Our experimental results indicate that Forsythiaside A also exhibits good inhibitory activity against *N. gonorrhoeae*. TH is a commonly used TCM known for its various medical properties, including antibacterial and antioxidant activities (Zhang et al., 2022). Its bioactive metabolites include flavonoids, polysaccharides, terpenoids, phenolic metabolites, taraxanthin, saponins, and other bioactive substances (Xie et al., 2022). TH demonstrates strong broad-spectrum antibacterial activity against *S. aureus*, *Escherichia coli* (*E. coli*), and other pathogens. In veterinary medicine, TH can serve as an available alternative to widely prohibited antibiotics (Mao et al., 2022). PH has gained attention as an effective TCM due to its rich array of bioactive ingredients. The natural products derived from it include flavonoids, which have been shown to impact the metabolism of carbohydrates and protein in *E. coli* and *S. aureus* (Okuda et al., 2021). The antibacterial mechanism of PH primarily involves acting on the cell membrane, leading to leakage of cellular contents and achieving bacteriostasis (Jalali and Ghasemzadeh Rahbardar, 2022). PS is a TCM recognized for its high nutritional and medicinal value. Its main active metabolites include triterpenoids, saponins, flavonoids, and lignans (Liu Z. et al., 2021). *In vitro* and *in vivo* studies have demonstrated that several monomer metabolites and crude extracts of PS possess activities such as anti-tumor, anti-inflammatory, antibacterial, and antiviral effects (Sun et al., 2021; Liu X. et al., 2022).

Five of the 13 TCMs (SR, DSR, SFR, CC and AR) exhibited no correlation with ceftriaxone regarding their MIC values. Among these, SR is a medicinal plant rich in various biologically active ingredients, including flavonoids, terpenoids, volatile oils and polysaccharides in its roots (Hu S. et al., 2022). The primary biochemical metabolites of SR are baicalin and baicalein, which have been demonstrated effective antibacterial properties against a variety of bacteria (Huang et al., 2022; Wang et al., 2022; Zeng et al., 2022). Additionally, studies have indicated that the combination of SR and CR produces a significant synergistic or additive bacteriostatic effect on against carbapenem-resistant *Klebsiella pneumoniae* (*K. pneumoniae*) *in vitro* (Jiang et al., 2020). In this study, SR exhibited MIC values ranging from ≤8 to >64 mg/mL and showed correlations with spectinomycin and azithromycin. Notably, among 18 azithromycin-resistant isolates, 83.3% had SR MIC values ≤16 mg/mL, suggesting that SR could serve as a potential therapeutic agent for azithromycin-resistant gonococcal infections. For DSR, its bioactive metabolites, primarily steroidal saponins, are responsible for its pharmacological effects (Guo et al., 2016). The MIC_10_ of DSR is 32 mg/mL, indicating a correlation between DSR MICs and azithromycin MICs, which suggests that DSR exhibits cross-resistance to azithromycin. DSR and BR are commonly combined to treat gonorrhea. This study investigated the antibacterial effect of this combination *in vitro*, revealing that the combination could effectively reduce the MIC by 52.9% and 98.6% compared to each drug used alone. SFR and its extracts primarily contain oxymatrine, matrine and flavonoids, all of which exhibit broad-spectrum antibacterial activity (Liang J. J. et al., 2022; Lin et al., 2022). These metabolites have shown considerable inhibitory effects against *S. aureus*, *Staphylococcus epidermidis* (*S. epidermidis*), *E. coli* and other pathogens (Oh et al., 2011; Chan et al., 2012; Tajima et al., 2016). CC is a well-known TCM recognized for its heat-clearing properties. The main metabolites of CC include terpenoids, phenylpropanoids, and glycosides. Modern research confirms that CC possesses a wide range of pharmacological effects, including antibacterial, anti-inflammatory, antiviral, and anticancer properties (Liu S. et al., 2021). AR has traditionally been used in TCM to treat gastrointestinal disorders and contains various metabolites, including triterpenoids, flavonoids, phenols, lactones, and alkaloids (Cao et al., 2022; Kim and Kim, 2022). Sesquiterpenoids are particularly notable therapeutic metabolites found in the rhizome of *Atractylodes Lancea* (Thunb.) DC. The MIC ranges for SFR, CC and AR are quite similar, at 8 to >64 mg/mL, 16–64 mg/mL, and 16 to >64 mg/mL, respectively, with MIC_90_ values are 32, 32 and 64 mg/mL, respectively. Correlation analysis of the MIC values indicates that SFR, CC, and AR all correlate with spectinomycin and azithromycin, suggesting an overlap in resistance genes among these three TCMs and the two antibiotics.

Only two of the 13 TCMs, BR and CFR, were found to be associated with all three antibiotics. BR is a botanical drug with strong pharmacological effects and is widely distributed (Qiao et al., 2022). The main bioactive ingredients of BR are saikosaponins (SSs), which have the potential to enhance the body’s immune function and possess anti-inflammatory, antioxidant, and immunostimulatory effects (Li et al., 2018; Cheng et al., 2022). *In vitro* studies have demonstrated that SSs monomers exhibit certain antibacterial effects when administered alone and can have synergistic or additive effects when combined with tetracycline (Higashitani et al., 1989). CFR, a TCM from the composite family, has been widely used in practices. The rhizome of this plant is rich in triterpenoid glycosides and exhibits significant antitumor activities both *in vitro* and *in vivo* (Wu et al., 2016). The MICs of BR and CFR correlated with those of ceftriaxone, azithromycin, and spectinomycin. However, these two TCMs may not be suitable for use alone in treating gonococcal infections due to the potential for cross-resistance.

## 5 Conclusion

This study is the first to evaluate the MICs of 13 TCMs against *N. gonorrhoeae* isolates from four Chinese provinces, analyze their correlation with clinical antibiotics to predict potential cross-resistance, and explore their potentially active chemical metabolites. The results indicate that CR, PCC, FF, TH, and SR exhibit good antibacterial activity against *N. gonorrhoeae* and may serve as promising candidates for treating gonococcal infections. The efficacy of PH, PS, DSR, BR, CC, CFR, AR, and SFR require further investigation. In China, azithromycin is not recommended as monotherapy for gonorrhea. Although SR exhibits cross-resistance with azithromycin, it shows lower MICs against azithromycin-resistant strains. TCMs have complex metabolites, typically containing multiple active ingredients with marked differences in content. Common antibacterial metabolites include alkaloids, flavonoids, saponins, quinones, polysaccharides, polyphenols, and terpenoids, all of which possess inherent antibacterial effects. While these TCMs are rarely used clinically to treat gonococcal infections and susceptibility data on gonococci to TCMs remain limited, an increasing number of patients are experiencing ceftriaxone resistance or persistent gonorrhea, which may necessitate the clinical use of TCMs.

Moreover, the pharmacokinetics of some TCMs have not been fully elucidated, as existing data are primarily based on *in vitro* experiments. The specific *in vivo* efficacy requires further investigation. Thus, additional research is needed to explore the phytochemical profiles of the 13 TCMs extracts using more sensitive analytical methods. It is essential to identify their antibacterial active metabolites, elucidate potential mechanisms of antibacterial action, and assess *in vivo* toxicity before considering them as new potential alternative agents for treating gonorrhea.

## Data Availability

The original contributions presented in the study are included in the article/Supplementary Material, further inquiries can be directed to the corresponding author.
