# Supplementary material for: Effect of 13 traditional Chinese medicine drug preparations against Neisseria gonorrhoeae
Source: Front Pharmacol. 2025 Nov 12;16:1694041. doi: 10.3389/fphar.2025.1694041 (PMC12647010; doi:10.3389/fphar.2025.1694041)
Supplement: Supplementary file 1 [file Supplementaryfile1.docx]

**SUPPLEMENTARY MATERIAL**


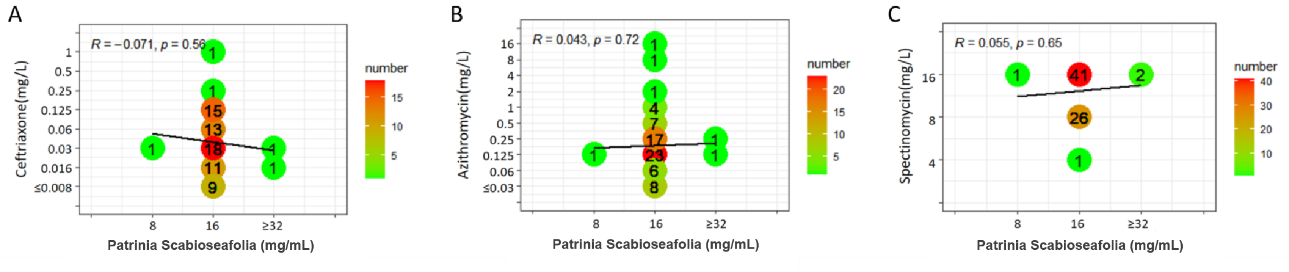


Figure S1 Correlation between MICs of Patrinia Scabioseafolia and ceftriaxone, spectinomycin, and azithromycin.


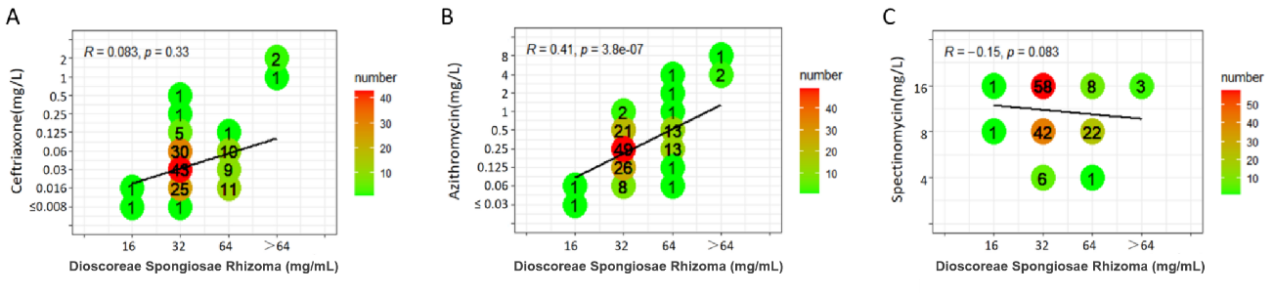


Figure S2 Correlation between MICs of Dioscoreae Spongiosae Rhizoma and ceftriaxone, spectinomycin, and azithromycin.


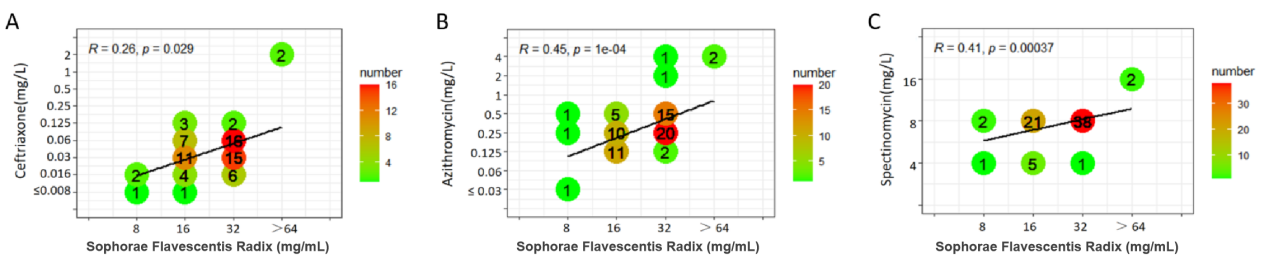


Figure S3 Correlation between MICs of Sophorae Flavescentis Radix and ceftriaxone, spectinomycin, and azithromycin.


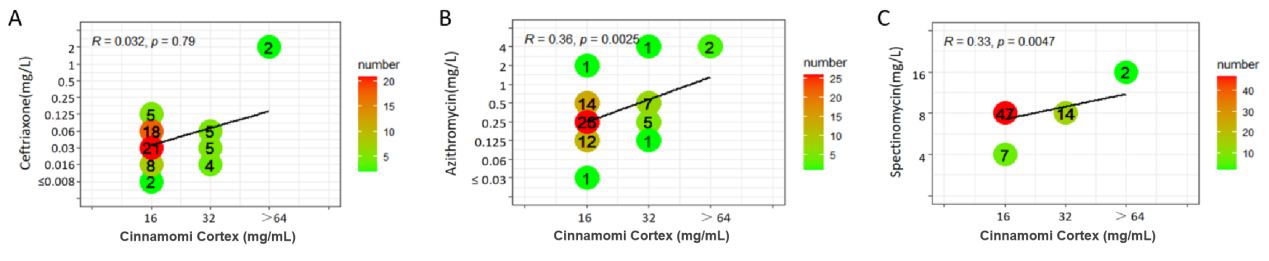


Figure S4 Correlation between MICs of Cinnamomi Cortex and ceftriaxone, spectinomycin, and azithromycin.


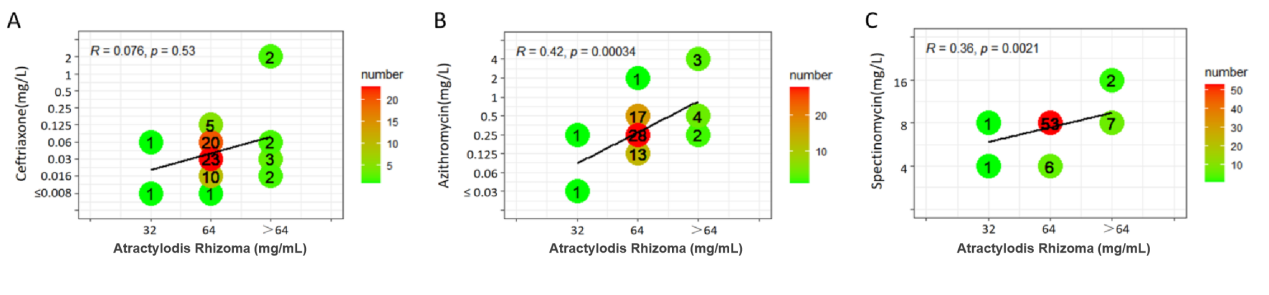


Figure S5 Correlation between MICs of Atractylodis Rhizoma and ceftriaxone, spectinomycin, and azithromycin.


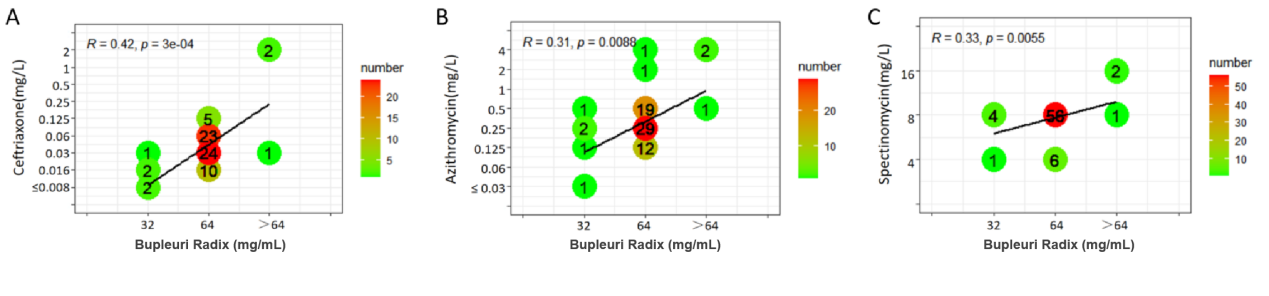


Figure S6 Correlation between MICs of Bupleuri Radix and ceftriaxone, spectinomycin, and azithromycin.


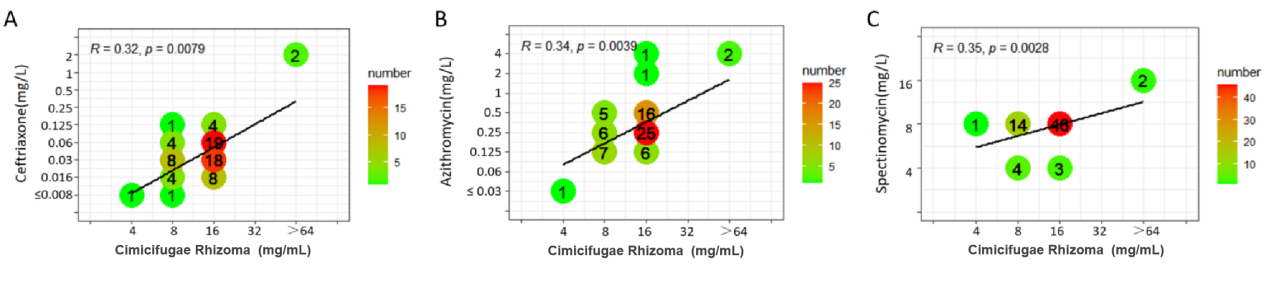


Figure S7 Correlation between MICs of Cimicifugae Rhizoma and ceftriaxone, spectinomycin, and azithromycin.


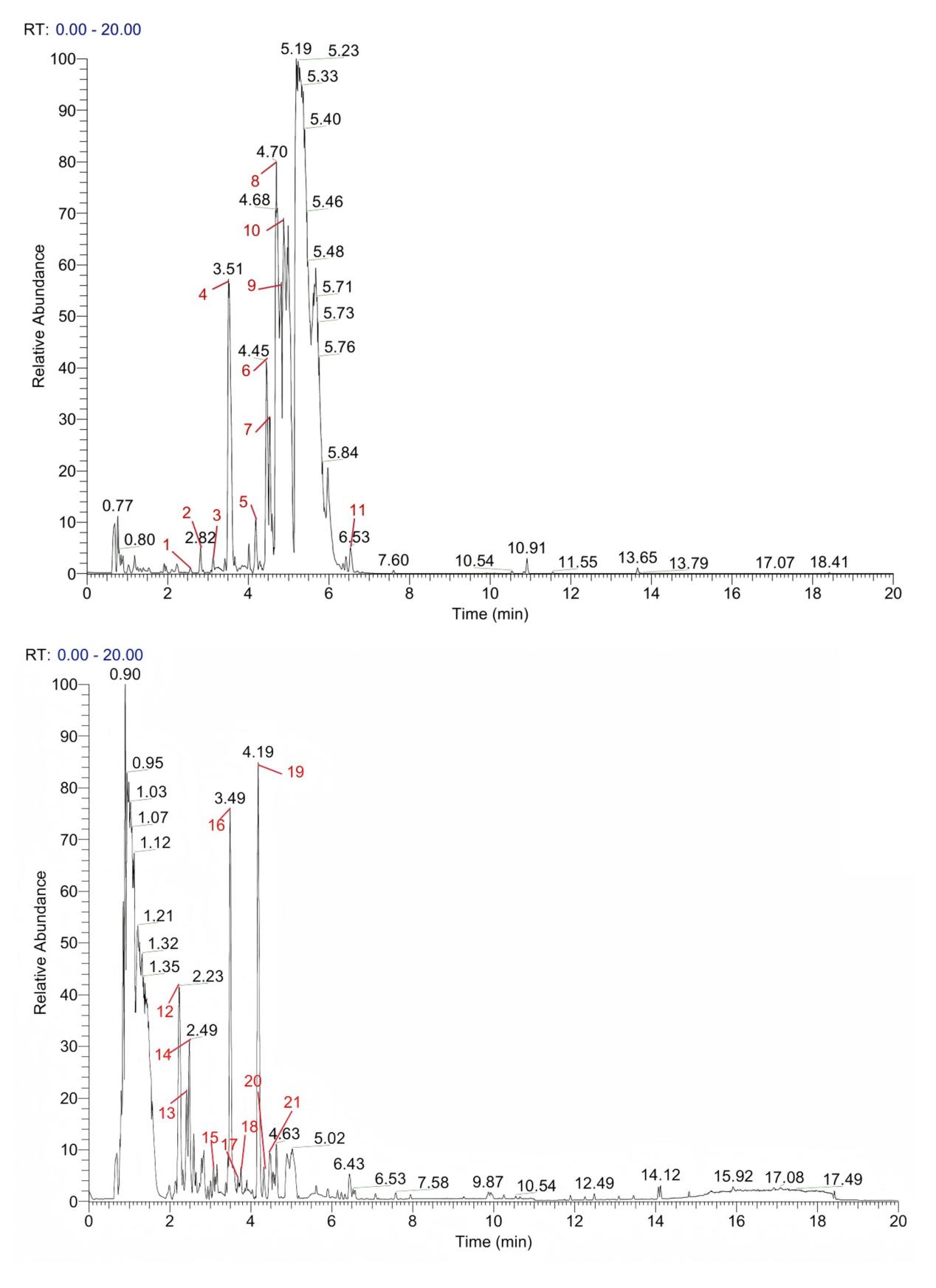


Figure S8 Base peak chromatogram (BPC) of Coptidis Rhizoma (CR) in positive ion mode (top figure) and negative ion mode (bottom figure).


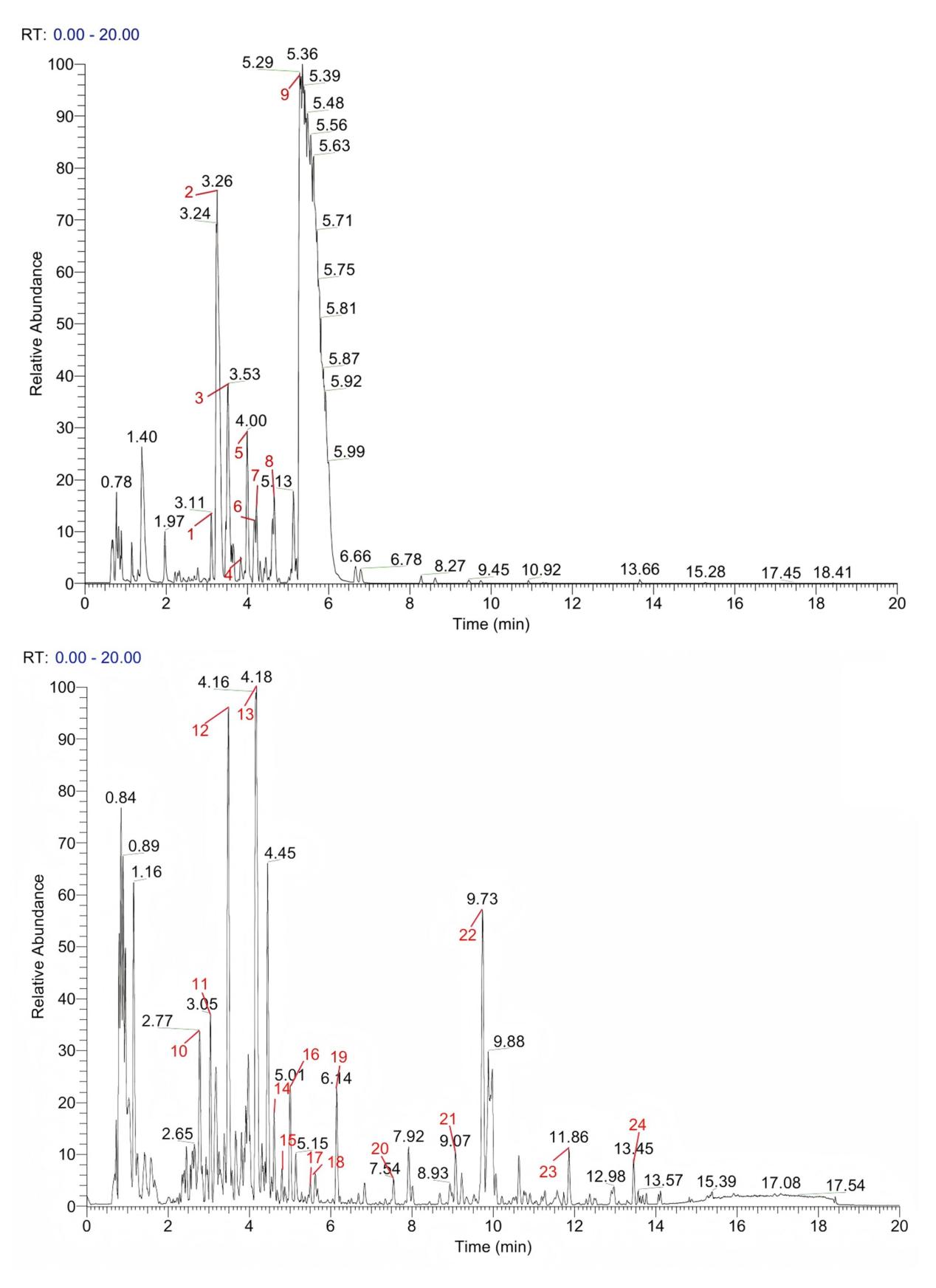


Figure S9 Base peak chromatogram (BPC) of Phellodendri Chinensis Cortex (PCC) in positive ion mode (top figure) and negative ion mode (bottom figure).

Table S1 The relevant parameters for preparation and identification of 13 TCM granules under the framework of national standard.

| **TCM ^a^** | **Preparation** | **Identification** | | | |
| --- | --- | --- | --- | --- | --- |
|  | **DER ^b^** | **ES ^c^** | **SMR ^d^** | **ED ^e^** | **ET ^f^** |
| Portulacae Herba (PH) | 1:4 | Ethyl alcohol | 40:1 | 20 min | RT ^g^ |
| Patrinia Scabiosaefolia (PS) | 1:7.5 | Ethyl acetate | 40:1 | 20 min | RT |
| Dioscoreae Spongiosae Rhizoma (DSR) | 1:4.3 | Ethyl acetate | 20:1 | 15 min | RT |
| Coptidis Rhizoma (CR) | 1:4.5 | Methanol | 250:1 | 30 min | RT |
| Bupleuri Radix (BR) | 1:4 | Methanol | 40:1 | 10 min | RT |
| Phellodendri Chinensis Cortex (PCC) | 1:5 | 1% acetic acid in methanol (v/v) | 40:1 | 20 min | 60 ℃ |
| Taraxaci Herba (TH) | 1:3.7 | 5% formic acid in methanol (v/v) | 20:1 | 20 min | RT |
| Forsythiae Fructus (FF) | 1:3.3 | Methanol | 25:1 | 20 min | RT |
| Scutellariae Radix (SR) | 1:2.2 | Methanol | 40:1 | 30 min | RT |
| Cinnamommi Cortex (CC) | 1:5.5 | Diethyl ether | 10:1 | 3 min | RT |
| Cimicifugae Rhizoma (CFR) | 1:5 | Ethyl alcohol | 200:1 | 30 min | RT |
| Atractylodis Rhizoma (AR) | 1:2.7 | Methanol | 20:1 | 15 min | RT |
| Sophorae Flavescentis Radix (SFR) | 1:5 | 1.2 % aqueous ammonia in methanol (v/v) | 50.6:1 | 30 min | RT |

^a^ TCM: Traditional Chinese Medicine.

^b^ DER: Drug to Genuine Extract Ratio.

^c^ ES: Extraction Solvent.

^d^ SMR: Solvent-to-Material Ratio, volume of solvent (mL): mass of material (g).

^e^ ED: Extraction Duration.

^f^ ET: Extraction Temperature.

^g^ RT: Room temperature.

Table S2 Peak-labeled identification results of metabolites in the base peak chromatogram (BPC) of Coptidis Rhizoma (CR).

| **No** | **m/z** | **RT/min** | **ppm** | **Adduct** | **Score** | **Metabolite Name** | **Super Class** |
| --- | --- | --- | --- | --- | --- | --- | --- |
| 1 | 300.1592 | 2.58 | 0.7 | [M+H]+ | 0.9737 | Codeine | Tyrosine alkaloids |
| 2 | 314.0656 | 2.83 | 8.1 | [M+H]+ | 0.8676 | * | NA |
| 3 | 330.0969 | 3.14 | 0.9 | [M+H]+ | 0.8889 | 4’, 6’-Dimethoxy-2’-hydroxy-3-nitrochalcone | Flavonoids |
| 4 | 342.1696 | 3.54 | 1.5 | [M]+ | 0.9399 | Magnoflorine | Tyrosine alkaloids |
| 5 | 177.0541 | 4.2 | 0.7 | [M+H-C₁₁H₁₂O₇]+ | 0.9897 | Cimicifuga acid A | Phenylpropanoids (C6-C3) |
| 6 | 322.107 | 4.47 | 0.5 | [M+H]+ | 0.9963 | Berberrubine | Tyrosine alkaloids |
| 7 | 324.1225 | 4.55 | 2.0 | [M]+ | 0.9416 | Demethyleneberberine | Tyrosine alkaloids |
| 8 | 320.0913 | 4.71 | 0.0 | [M]+ | 0.9955 | Coptisine | Tyrosine alkaloids |
| 9 | 336.1226 | 4.82 | 1.3 | [M]+ | 0.9992 | Epiberberine | Tyrosine alkaloids |
| 10 | 338.1381 | 4.88 | 0.2 | [M]+ | 0.9987 | Neprotin | Tyrosine alkaloids |
| 11 | 350.1384 | 6.55 | 5.6 | [M+Na]+ | 0.8598 | 6-Acetylmorphine | Tyrosine alkaloids |
| 12 | 359.0981 | 2.23 | 1.2 | [M-H]- | 0.876 | 1-O-(3-Hydroxy-4,5-dimethoxybenzoyl) hexopyranose | Phenolic acids (C6-C1) |
| 13 | 315.072 | 2.42 | 3.8 | [M-H]- | 0.909 | Benzoic acid + 2O, O-Hex | Phenolic acids (C6-C1) |
| 14 | 197.0449 | 2.48 | 2.5 | [M-H]- | 0.7721 | (R)-3-(3,4-Dihydroxyphenyl) lactate | NA |
| 15 | 181.0499 | 3.08 | 4.1 | [M-H]- | 0.9936 | p-Hydroxyphenyllactic acid | NA |
| 16 | 367.103 | 3.49 | 1.2 | [M-H]- | 0.991 | 5-Feruloylquinic acid | Phenylpropanoids (C6-C3) |
| 17 | 215.0821 | 3.69 | 2.5 | [M-H]- | 0.7932 | 5-Phenethyl-1H-pyrazole-3-carboxylic acid | NA |
| 18 | 521.2024 | 3.76 | 0.3 | [M-H]- | 0.9159 | ((2R,3R,4S)-6-Hydroxy-4-(4-(hydroxymethyl)-7-methoxy-1,2,3,4-tetrahydronaphthalen-2-yl) methyl β-D-glucopyranoside | Lignans |
| 19 | 367.103 | 4.19 | 3.2 | [M-H]- | 0.8989 | Methyl 5-O-caffeoylquinate | Phenylpropanoids (C6-C3) |
| 20 | 567.208 | 4.33 | 0.4 | [M+HCOO]- | 0.8403 | Indigoticoside A | Lignans |
| 21 | 322.1082 | 4.48 | 1.0 | [M-H]- | 0.7683 | 1-Allyl-3-keto-2-(3-methoxyphenyl) isoindoline-4-carboxylic acid | Anthranilic acid alkaloids |

* Compound#2 may have been misannotated, so it has been removed after manual review.

Table S3 Peak-labeled identification results of metabolites in the base peak chromatogram (BPC) of Phellodendri Chinensis Cortex (PCC).

| **No** | **m/z** | **RT/min** | **ppm** | **Adduct** | **Score** | **Metabolite Name** | **Super Class** |
| --- | --- | --- | --- | --- | --- | --- | --- |
| 1 | 298.1435 | 3.12 | 1.2 | [M+H]+ | 0.9667 | * | NA |
| 2 | 342.1696 | 3.27 | 0.2 | [M]+ | 0.9982 | Phellodendrine | Tyrosine alkaloids |
| 3 | 342.1697 | 3.53 | 0.7 | [M]+ | 0.9417 | Magnoflorine | Tyrosine alkaloids |
| 4 | 312.1228 | 3.85 | 0.7 | [M+H-H₂O]+ | 0.9731 | N-trans-Feruloyloctopamine | Phenylpropanoids (C6-C3) |
| 5 | 314.1748 | 4.01 | 1.1 | [M+H]+ | 0.9098 | Codethyline | Tyrosine alkaloids |
| 6 | 177.0545 | 4.19 | 0.5 | [M+H-C₁₁H₁₂O₇]+ | 0.9895 | Cimicifuga acid A | Phenylpropanoids (C6-C3) |
| 7 | 356.1853 | 4.24 | 0.8 | [M+H]+ | 0.8668 | Tetrahydropalmatin | Tyrosine alkaloids |
| 8 | 352.1178 | 4.68 | 1.5 | [M+H]+ | 0.8708 | Oxoglaucine | Tyrosine alkaloids |
| 9 | 336.1227 | 5.31 | 0.1 | [M]+ | 0.989 | Majarine | Tyrosine alkaloids |
| 10 | 353.0876 | 2.79 | 0.3 | [M-H-C₉H₆O₃]- | 0.9738 | Cynarin | Phenylpropanoids (C6-C3) |
| 11 | 431.1555 | 3.05 | 0.6 | [M-H]- | 0.9554 | Osmanthuside H | Phenylethanoids (C6-C2) |
| 12 | 367.1032 | 3.49 | 1.2 | [M-H]- | 0.9861 | 5-Feruloylquinic acid | Phenylpropanoids (C6-C3) |
| 13 | 367.1031 | 4.17 | 2.2 | [M-H]- | 0.9707 | 1,3,5-Trihydroxy-4-[(E)-3-(3-methoxyphenyl) acryloyl] oxycyclohexanecarboxylic acid | Phenylpropanoids (C6-C3) |
| 14 | 567.2082 | 4.61 | 0.4 | [M+HCOO]- | 0.858 | 5-(3-(Hydroxymethyl)-5-(3-hydroxypropyl)-7-methoxy-2,3-dihydro-1-benzofuran-2-yl)-2-methoxyphenyl hexopyranoside | Lignans |
| 15 | 163.0391 | 4.8 | 4.6 | [M-H]- | 0.9994 | Coumaric acid | Phenylpropanoids (C6-C3) |
| 16 | 193.05 | 5.0 | 3.0 | [M-H-C₁₅H₂₀O₉]- | 0.9925 | Picroside III | Monoterpenoids |
| 17 | 207.0658 | 5.51 | 2.3 | [M-H]- | 0.9867 | Sinapoyl aldehyde | Phenylpropanoids (C6-C3) |
| 18 | 177.055 | 5.62 | 4.1 | [M-H]- | 0.9978 | Coniferyl aldehyde | Phenylpropanoids (C6-C3) |
| 19 | 187.0969 | 6.16 | 2.9 | [M-H]- | 0.9969 | Azelaic acid | Fatty Acids and Conjugates |
| 20 | 201.1127 | 7.55 | 2.3 | [M-H]- | 0.9803 | Sebacic acid | Fatty Acids and Conjugates |
| 21 | 327.2175 | 9.07 | 3.9 | [M-H]- | 0.987 | FA 18:2+3O | Octadecanoids |
| 22 | 515.1919 | 9.74 | 0.4 | [M+HCO₂]- | 0.9893 | Evodin | Triterpenoids |
| 23 | 311.2227 | 11.87 | 0.2 | [M-H]- | 0.9903 | (9Z,12E)-15,16-Dihydroxyoctadeca-9,12-dienoic acid | Octadecanoids |
| 24 | 295.2275 | 13.46 | 1.2 | [M-H]- | 0.9932 | 12(13)-EpOME | Fatty Acids and Conjugates |

* Compound#1 may have been misannotated, so it has been removed after manual review.

Table S4 Antimicrobial susceptibility testing results for clinical antibiotics and 18 selected representative metabolites in CR, PCC, SR, TH and FF.

| **Strain number** | **MIC (mg/mL)** | | | | | | | | | | | | | | | | | | | | |
| --- | --- | --- | --- | --- | --- | --- | --- | --- | --- | --- | --- | --- | --- | --- | --- | --- | --- | --- | --- | --- | --- |
|  | **Antibiotics*** | | | **CR** | | | **PCC** | | | **SR** | | | | **TH** | | **FF** | | | | | |
|  | **CRO** | **CFM** | **AZM** | **Magnoflorine** | **Epiberberine** | **Coptisine** | **Phellodendrine** | **Berberine** | **5-Feruloylquinic acid** | **Norwogonin** | **Baicalein** | **Wogonoside** | **Skullcapflavone II** | **Caffeic acid (mg/L)** | **Chicoric acid** | **Forsythiaside A** | **Forsythoside B** | **Forsythoside E** | **Forsythoside I** | **Gibberellic acid** | **Jatrorrhizine** |
| L633 | 0.5 | 2 | 0.06 | ＞4.6 | ＞3.7 | ＜0.03 | ＞3.8 | 0.45 | 3.68 | ＞2.7 | ＞2.7 | ＞4.6 | ＞3.74 | ＞1.8 | 0.12 | 0.1 | 3.78 | ＞4.6 | ＞6.2 | ＞3.4 | ＞3.7 |
| L160 | 0.06 | 0.06 | 0.25 | ＞4.6 | ＞3.7 | ＜0.03 | ＞3.8 | ＜0.075 | ＜0.03 | ＞2.7 | ＞2.7 | ＞4.6 | ＜0.03 | ＞1.8 | ＜0.0075 | 0.05 | ＜0.06 | ＞4.6 | ＞6.2 | ＞3.4 | ＞3.7 |
| L95 | 0.5 | 2 | 0.25 | ＞4.6 | ＞3.7 | ＜0.03 | ＞3.8 | 0.22 | 1.84 | ＞2.7 | ＞2.7 | ＞4.6 | 1.87 | ＞1.8 | 0.06 | 0.2 | 3.78 | ＞4.6 | ＞6.2 | ＞3.4 | ＞3.7 |
| L201 | 1 | 2 | 0.25 | ＞4.6 | ＞3.7 | ＜0.03 | ＞3.8 | ＜0.075 | ＜0.03 | ＞2.7 | ＞2.7 | ＞4.6 | ＜0.03 | ＞1.8 | ＜0.0075 | ＜0.05 | ＜0.06 | ＞4.6 | ＞6.2 | ＞3.4 | ＞3.7 |
| L462 | ＜0.06 | 0.06 | 0.125 | ＞4.6 | ＞3.7 | ＜0.03 | ＞3.8 | ＜0.075 | ＜0.03 | ＞2.7 | ＞2.7 | ＞4.6 | ＜0.03 | ＞1.8 | ＜0.0075 | ＜0.05 | ＜0.06 | ＞4.6 | ＞6.2 | ＞3.4 | ＞3.7 |
| L470 | 0.5 | 2 | 0.25 | ＞4.6 | ＞3.7 | ＜0.03 | ＞3.8 | ＜0.075 | 0.46 | ＞2.7 | ＞2.7 | ＞4.6 | ＜0.03 | ＞1.8 | ＜0.0075 | 0.05 | 0.06 | ＞4.6 | ＞6.2 | ＞3.4 | ＞3.7 |
| L662 | 0.5 | 2 | 0.25 | ＞4.6 | ＞3.7 | ＜0.03 | ＞3.8 | ＜0.075 | ＜0.03 | ＞2.7 | ＞2.7 | ＞4.6 | ＜0.03 | ＞1.8 | ＜0.0075 | ＜0.05 | ＜0.06 | ＞4.6 | ＞6.2 | ＞3.4 | ＞3.7 |
| L163 | ＜0.06 | ＜0.06 | 0.25 | ＞4.6 | ＞3.7 | ＜0.03 | ＞3.8 | 0.22 | 1.84 | ＞2.7 | ＞2.7 | ＞4.6 | 1.87 | ＞1.8 | 0.06 | 3.12 | 3.78 | ＞4.6 | ＞6.2 | ＞3.4 | ＞3.7 |
| L576 | 0.5 | 2 | 1 | ＞4.6 | ＞3.7 | ＜0.03 | ＞3.8 | ＜0.075 | 0.46 | ＞2.7 | ＞2.7 | ＞4.6 | ＜0.03 | ＞1.8 | ＜0.0075 | 0.05 | 0.06 | ＞4.6 | ＞6.2 | ＞3.4 | ＞3.7 |
| L171 | ＜0.06 | 0.06 | 0.125 | ＞4.6 | ＞3.7 | 0.03 | ＞3.8 | 0.22 | 3.68 | ＞2.7 | ＞2.7 | ＞4.6 | 1.87 | ＞1.8 | 0.24 | 3.12 | 3.78 | ＞4.6 | ＞6.2 | ＞3.4 | ＞3.7 |
| L178 | 0.06 | 0.06 | 0.25 | ＞4.6 | ＞3.7 | 1.04 | ＞3.8 | 0.9 | 3.68 | ＞2.7 | ＞2.7 | ＞4.6 | 3.74 | ＞1.8 | 0.95 | 6.25 | 7.57 | ＞4.6 | ＞6.2 | ＞3.4 | ＞3.7 |
| L614 | 0.125 | 2 | 0.25 | ＞4.6 | ＞3.7 | 0.03 | ＞3.8 | 0.22 | 1.84 | ＞2.7 | ＞2.7 | ＞4.6 | 1.87 | ＞1.8 | 0.24 | 1.56 | 3.78 | ＞4.6 | ＞6.2 | ＞3.4 | ＞3.7 |
| L93 | 0.06 | 0.06 | 0.25 | ＞4.6 | ＞3.7 | ＜0.03 | ＞3.8 | ＜0.075 | 0.46 | ＞2.7 | ＞2.7 | ＞4.6 | 0.47 | ＞1.8 | ＜0.0075 | 0.1 | 0.06 | ＞4.6 | ＞6.2 | ＞3.4 | ＞3.7 |
| L641 | 0.5 | 2 | 0.25 | ＞4.6 | ＞3.7 | ＜0.03 | ＞3.8 | ＜0.075 | ＜0.03 | ＞2.7 | ＞2.7 | ＞4.6 | ＜0.03 | ＞1.8 | ＜0.0075 | 0.05 | 0.06 | ＞4.6 | ＞6.2 | ＞3.4 | ＞3.7 |
| L636 | 0.5 | 2 | 0.25 | ＞4.6 | ＞3.7 | ＜0.03 | ＞3.8 | ＜0.075 | ＜0.03 | ＞2.7 | ＞2.7 | ＞4.6 | ＜0.03 | ＞1.8 | ＜0.0075 | 0.05 | 0.06 | ＞4.6 | ＞6.2 | ＞3.4 | ＞3.7 |
| L153 | 0.06 | 0.5 | 0.25 | ＞4.6 | ＞3.7 | ＜0.03 | ＞3.8 | 0.22 | 1.84 | ＞2.7 | ＞2.7 | ＞4.6 | 1.87 | ＞1.8 | 0.03 | 1.56 | 3.78 | ＞4.6 | ＞6.2 | ＞3.4 | ＞3.7 |
| L538 | 0.5 | 2 | ＞16 | ＞4.6 | ＞3.7 | 0.03 | ＞3.8 | 0.22 | 1.84 | ＞2.7 | ＞2.7 | ＞4.6 | 1.87 | ＞1.8 | 0.06 | 3.12 | 3.78 | ＞4.6 | ＞6.2 | ＞3.4 | ＞3.7 |
| L175 | ＜0.06 | 0.06 | 1 | ＞4.6 | ＞3.7 | ＜0.03 | ＞3.8 | ＜0.075 | ＜0.03 | ＞2.7 | ＞2.7 | ＞4.6 | ＜0.03 | ＞1.8 | ＜0.0075 | 0.05 | ＜0.06 | ＞4.6 | ＞6.2 | ＞3.4 | ＞3.7 |
| L176 | 0.06 | 0.06 | 0.06 | ＞4.6 | ＞3.7 | ＜0.03 | ＞3.8 | 0.22 | 1.84 | ＞2.7 | ＞2.7 | ＞4.6 | 1.87 | ＞1.8 | 0.03 | 3.12 | 3.78 | ＞4.6 | ＞6.2 | ＞3.4 | ＞3.7 |
| L607 | 0.5 | 2 | 0.25 | ＞4.6 | ＞3.7 | ＜0.03 | ＞3.8 | ＜0.075 | 0.46 | ＞2.7 | ＞2.7 | ＞4.6 | 0.23 | ＞1.8 | ＜0.0075 | 0.05 | 0.06 | ＞4.6 | ＞6.2 | ＞3.4 | ＞3.7 |
| L91 | 0.5 | 2 | 0.5 | ＞4.6 | ＞3.7 | 1.04 | ＞3.8 | 0.9 | >3.68 | ＞2.7 | ＞2.7 | ＞4.6 | 3.74 | ＞1.8 | 0.95 | 6.25 | 7.57 | ＞4.6 | ＞6.2 | ＞3.4 | ＞3.7 |
| L398 | 0.5 | 2 | 0.25 | ＞4.6 | ＞3.7 | 0.26 | ＞3.8 | 0.45 | 3.68 | ＞2.7 | ＞2.7 | ＞4.6 | 1.87 | ＞1.8 | 0.24 | 6.25 | 7.57 | ＞4.6 | ＞6.2 | ＞3.4 | ＞3.7 |
| L529 | 0.5 | 2 | 1 | ＞4.6 | ＞3.7 | ＜0.03 | ＞3.8 | ＜0.075 | 0.92 | ＞2.7 | ＞2.7 | ＞4.6 | ＜0.03 | ＞1.8 | ＜0.0075 | 0.05 | 0.06 | ＞4.6 | ＞6.2 | ＞3.4 | ＞3.7 |
| L101 | 0.06 | 0.5 | 2 | ＞4.6 | ＞3.7 | ＜0.03 | ＞3.8 | ＜0.075 | 0.92 | ＞2.7 | ＞2.7 | ＞4.6 | 0.47 | ＞1.8 | ＜0.0075 | 0.78 | 1.89 | ＞4.6 | ＞6.2 | ＞3.4 | ＞3.7 |
| L103 | 0.5 | 2 | 0.25 | ＞4.6 | ＞3.7 | ＜0.03 | ＞3.8 | ＜0.075 | 1.84 | ＞2.7 | ＞2.7 | ＞4.6 | ＜0.03 | ＞1.8 | ＜0.0075 | 0.05 | 0.06 | ＞4.6 | ＞6.2 | ＞3.4 | ＞3.7 |
| L630 | 1 | 4 | 0.125 | ＞4.6 | ＞3.7 | 1.04 | ＞3.8 | 0.45 | 3.68 | ＞2.7 | ＞2.7 | ＞4.6 | 3.74 | ＞1.8 | 0.95 | 6.25 | 7.57 | ＞4.6 | ＞6.2 | ＞3.4 | ＞3.7 |
| L446 | 0.5 | 2 | 0.5 | ＞4.6 | ＞3.7 | ＜0.03 | ＞3.8 | ＜0.075 | ＜0.03 | ＞2.7 | ＞2.7 | ＞4.6 | ＜0.03 | ＞1.8 | ＜0.0075 | ＜0.05 | ＜0.06 | ＞4.6 | ＞6.2 | ＞3.4 | ＞3.7 |
| L423 | 1 | 2 | 1 | ＞4.6 | ＞3.7 | ＜0.03 | ＞3.8 | ＜0.075 | ＜0.03 | ＞2.7 | ＞2.7 | ＞4.6 | ＜0.03 | ＞1.8 | ＜0.0075 | ＜0.05 | ＜0.06 | ＞4.6 | ＞6.2 | ＞3.4 | ＞3.7 |
| L80 | 0.06 | 0.125 | 0.06 | ＞4.6 | ＞3.7 | ＜0.03 | ＞3.8 | ＜0.075 | 0.23 | ＞2.7 | ＞2.7 | ＞4.6 | 0.23 | ＞1.8 | ＜0.0075 | 0.05 | 0.95 | ＞4.6 | ＞6.2 | ＞3.4 | ＞3.7 |
| L217 | 0.5 | 2 | 1 | ＞4.6 | ＞3.7 | 0.26 | ＞3.8 | 0.45 | 3.68 | ＞2.7 | ＞2.7 | ＞4.6 | 1.87 | ＞1.8 | 0.24 | 6.25 | 7.57 | ＞4.6 | ＞6.2 | ＞3.4 | ＞3.7 |
| L104 | 0.5 | 2 | 0.25 | ＞4.6 | ＞3.7 | ＜0.03 | ＞3.8 | ＜0.075 | 0.23 | ＞2.7 | ＞2.7 | ＞4.6 | ＜0.03 | ＞1.8 | ＜0.0075 | 0.05 | 0.06 | ＞4.6 | ＞6.2 | ＞3.4 | ＞3.7 |
| L588 | 1 | 4 | 0.25 | ＞4.6 | ＞3.7 | ＜0.03 | ＞3.8 | ＜0.075 | 0.46 | ＞2.7 | ＞2.7 | ＞4.6 | ＜0.03 | ＞1.8 | ＜0.0075 | 0.05 | 0.06 | ＞4.6 | ＞6.2 | ＞3.4 | ＞3.7 |
| L147 | ＜0.06 | ＜  0.06 | 0.25 | ＞4.6 | ＞3.7 | 1.04 | ＞3.8 | 0.9 | >3.68 | ＞2.7 | ＞2.7 | ＞4.6 | 3.74 | ＞1.8 | 0.95 | 6.25 | 7.57 | ＞4.6 | ＞6.2 | ＞3.4 | ＞3.7 |
| L346 | 0.5 | 4 | 0.25 | ＞4.6 | ＞3.7 | ＜0.03 | ＞3.8 | ＜0.075 | 0.92 | ＞2.7 | ＞2.7 | ＞4.6 | 0.47 | ＞1.8 | ＜0.0075 | 0.05 | 0.06 | ＞4.6 | ＞6.2 | ＞3.4 | ＞3.7 |
| L412 | 0.125 | 1 | ＜0.06 | ＞4.6 | ＞3.7 | ＜0.03 | ＞3.8 | 0.015 | 1.84 | ＞2.7 | ＞2.7 | ＞4.6 | 1.87 | ＞1.8 | 0.0075 | 1.56 | 0.95 | ＞4.6 | ＞6.2 | ＞3.4 | ＞3.7 |
| L342 | 0.06 | 0.125 | 0.125 | ＞4.6 | ＞3.7 | ＜0.03 | ＞3.8 | 0.22 | 0.23 | ＞2.7 | ＞2.7 | ＞4.6 | 0.90.47 | ＞1.8 | ＜0.0075 | 0.1 | 0.95 | ＞4.6 | ＞6.2 | ＞3.4 | ＞3.7 |
| L92 | 0.5 | 2 | 0.25 | ＞4.6 | ＞3.7 | ＜0.03 | ＞3.8 | ＜0.075 | 0.46 | ＞2.7 | ＞2.7 | ＞4.6 | 0.47 | ＞1.8 | ＜0.0075 | 0.05 | 0.06 | ＞4.6 | ＞6.2 | ＞3.4 | ＞3.7 |
| L81 | 1 | 4 | 2 | ＞4.6 | ＞3.7 | 1.04 | ＞3.8 | 0.9 | >3.68 | ＞2.7 | ＞2.7 | ＞4.6 | 3.74 | ＞1.8 | 0.95 | 6.25 | 7.57 | ＞4.6 | ＞6.2 | ＞3.4 | ＞3.7 |
| L123 | 0.06 | 0.06 | 0.125 | ＞4.6 | ＞3.7 | ＜0.03 | ＞3.8 | ＜0.075 | 0.23 | ＞2.7 | ＞2.7 | ＞4.6 | ＜0.03 | ＞1.8 | ＜0.0075 | 0.05 | 0.06 | ＞4.6 | ＞6.2 | ＞3.4 | ＞3.7 |
| L700 | 1 | 4 | 0.125 | ＞4.6 | ＞3.7 | ＜0.03 | ＞3.8 | ＜0.075 | 0.23 | ＞2.7 | ＞2.7 | ＞4.6 | ＜0.03 | ＞1.8 | ＜0.0075 | 0.05 | 0.06 | ＞4.6 | ＞6.2 | ＞3.4 | ＞3.7 |
| L105 | 0.5 | 2 | 0.25 | ＞4.6 | ＞3.7 | ＜0.03 | ＞3.8 | ＜0.075 | 0.46 | ＞2.7 | ＞2.7 | ＞4.6 | ＜0.03 | ＞1.8 | ＜0.0075 | 0.05 | 0.06 | ＞4.6 | ＞6.2 | ＞3.4 | ＞3.7 |
| L215 | 0.5 | 2 | 0.25 | ＞4.6 | ＞3.7 | ＜0.03 | ＞3.8 | ＜0.075 | 1.84 | ＞2.7 | ＞2.7 | ＞4.6 | 0.90.47 | ＞1.8 | ＜0.0075 | 0.1 | 0.95 | ＞4.6 | ＞6.2 | ＞3.4 | ＞3.7 |
| L243 | 0.5 | 2 | 0.25 | ＞4.6 | ＞3.7 | ＜0.03 | ＞3.8 | ＜0.075 | ＜0.03 | ＞2.7 | ＞2.7 | ＞4.6 | ＜0.03 | ＞1.8 | ＜0.0075 | ＜0.05 | ＜0.06 | ＞4.6 | ＞6.2 | ＞3.4 | ＞3.7 |
| L314 | 0.5 | 2 | 0.25 | ＞4.6 | ＞3.7 | 0.26 | ＞3.8 | 0.45 | 3.68 | ＞2.7 | ＞2.7 | ＞4.6 | 3.74 | ＞1.8 | 0.12 | 6.25 | 7.57 | ＞4.6 | ＞6.2 | ＞3.4 | ＞3.7 |
| L592 | 0.5 | 2 | 0.125 | ＞4.6 | ＞3.7 | ＜0.03 | ＞3.8 | ＜0.075 | 0.46 | ＞2.7 | ＞2.7 | ＞4.6 | 0.23 | ＞1.8 | ＜0.0075 | 0.05 | 0.06 | ＞4.6 | ＞6.2 | ＞3.4 | ＞3.7 |
| L489 | 0.5 | 2 | 1 | ＞4.6 | ＞3.7 | ＜0.03 | ＞3.8 | ＜0.075 | 0.46 | ＞2.7 | ＞2.7 | ＞4.6 | 0.23 | ＞1.8 | ＜0.0075 | 0.05 | 0.06 | ＞4.6 | ＞6.2 | ＞3.4 | ＞3.7 |
| L471 | ＜0.06 | ＜0.06 | 0.5 | ＞4.6 | ＞3.7 | 4.17 | ＞3.8 | 0.9 | >3.68 | ＞2.7 | ＞2.7 | ＞4.6 | ＞3.74 | ＞1.8 | 0.95 | 6.25 | 7.57 | ＞4.6 | ＞6.2 | ＞3.4 | ＞3.7 |
| L128 | 1 | 2 | 1 | ＞4.6 | ＞3.7 | ＜0.03 | ＞3.8 | ＜0.075 | ＜0.03 | ＞2.7 | ＞2.7 | ＞4.6 | ＜0.03 | ＞1.8 | ＜0.0075 | ＜0.05 | ＜0.06 | ＞4.6 | ＞6.2 | ＞3.4 | ＞3.7 |
| L82 | 1 | 4 | 2 | ＞4.6 | ＞3.7 | 1.04 | ＞3.8 | 0.45 | 3.68 | ＞2.7 | ＞2.7 | ＞4.6 | 3.74 | ＞1.8 | 0.24 | 6.25 | 3.78 | ＞4.6 | ＞6.2 | ＞3.4 | ＞3.7 |
| WHO-O | ＜0.06 | ＜0.06 | 0.125 | ＞4.6 | ＞3.7 | 0.26 | ＞3.8 | 0.45 | 1.84 | ＞2.7 | ＞2.7 | ＞4.6 | 3.74 | ＞1.8 | 0.0075 | 3.12 | 7.57 | ＞4.6 | ＞6.2 | ＞3.4 | ＞3.7 |
| WHO-X | 2 | ＞4 | 0.125 | ＞4.6 | ＞3.7 | 1.04 | ＞3.8 | 0.45 | >3.68 | ＞2.7 | ＞2.7 | ＞4.6 | 3.74 | ＞1.8 | 0.24 | 6.25 | 7.57 | ＞4.6 | ＞6.2 | ＞3.4 | ＞3.7 |
| WHO-Y | 1 | 4 | 0.125 | ＞4.6 | ＞3.7 | ＜0.03 | ＞3.8 | ＜0.075 | ＜0.03 | ＞2.7 | ＞2.7 | ＞4.6 | 0.47 | ＞1.8 | ＜0.0075 | ＜0.05 | 1.89 | ＞4.6 | ＞6.2 | ＞3.4 | ＞3.7 |
| WHO-Z | 0.5 | 2 | 0.5 | ＞4.6 | ＞3.7 | ＞4.17 | ＞3.8 | ＞0.9 | >3.68 | ＞2.7 | ＞2.7 | ＞4.6 | ＞3.74 | ＞1.8 | ＞0.95 | ＞6.25 | ＞7.57 | ＞4.6 | ＞6.2 | ＞3.4 | ＞3.7 |

* CRO:Ceftriaxone; CFM: Cefixime; AZM: Azithromycin.
